# Immunoregulatory and neutrophil-like monocyte subsets with distinct single-cell transcriptomic signatures emerge following brain injury

**DOI:** 10.1186/s12974-024-03032-8

**Published:** 2024-02-03

**Authors:** Erwin K. Gudenschwager Basso, Jing Ju, Eman Soliman, Caroline de Jager, Xiaoran Wei, Kevin J. Pridham, Michelle L. Olsen, Michelle H. Theus

**Affiliations:** 1 Department of Biomedical Sciences and Pathobiology, Virginia Tech, 970 Washington Street SW, Life Sciences I, Rm 249 (MC0910), Blacksburg, VA 24061 USA; 2 Center for Engineered Health, Virginia Tech, Blacksburg, VA 24061 USA; 3 School of Neuroscience, Virginia Tech, Blacksburg, VA 24061 USA; 4 Translational, Biology, Medicine and Health Graduate Program, Virginia Tech, Roanoke, VA 24016 USA; 5 Department of Physiology and Aging, College of Medicine, University of Florida, Gainesville, FL USA

**Keywords:** Clodronate, Innate immunity, Traumatic brain injury, Neuroinflammation

## Abstract

Monocytes represent key cellular elements that contribute to the neurological sequela following brain injury. The current study reveals that trauma induces the augmented release of a transcriptionally distinct CD115^+^/Ly6C^hi^ monocyte population into the circulation of mice pre-exposed to clodronate depletion conditions. This phenomenon correlates with tissue protection, blood–brain barrier stability, and cerebral blood flow improvement. Uniquely, this shifted the innate immune cell profile in the cortical milieu and reduced the expression of pro-inflammatory Il6, IL1r1, MCP-1, Cxcl1, and Ccl3 cytokines. Monocytes that emerged under these conditions displayed a morphological and gene profile consistent with a subset commonly seen during emergency monopoiesis. Single-cell RNA sequencing delineated distinct clusters of monocytes and revealed a key transcriptional signature of Ly6C^hi^ monocytes enriched for *Apoe* and chitinase-like protein 3 (*Chil3*/Ym1), commonly expressed in pro-resolving immunoregulatory monocytes, as well as granule genes *Elane*,* Prtn3*,* MPO*, and *Ctsg* unique to neutrophil-like monocytes. The predominate shift in cell clusters included subsets with low expression of transcription factors involved in monocyte conversion, *Pou2f2*, *Na4a1,* and a robust enrichment of genes in the oxidative phosphorylation pathway which favors an anti-inflammatory phenotype. Transfer of this monocyte assemblage into brain-injured recipient mice demonstrated their direct role in neuroprotection. These findings reveal a multifaceted innate immune response to brain injury and suggest targeting surrogate monocyte subsets may foster tissue protection in the brain.

**Supplementary Information:**

The online version contains supplementary material available at 10.1186/s12974-024-03032-8.

## Introduction

The heterogeneity and plasticity of monocytes/macrophages are thought to play crucial roles in traumatic central nervous system (CNS) injuries [1–3]. They contribute to scar formation, tissue resolution, and acute neuronal death. Understanding and manipulating the different functions and behaviors of monocyte/macrophage subpopulations may offer potential therapeutic strategies for addressing the consequences of traumatic brain injuries. Monocytes are important sources of cytokines, chemokines, and oxidative stress molecules [4, 5]. Recent studies indicate that the complex milieu of cytokines in the surrounding environment influences the polarization of peripheral-derived monocytes/macrophages, resulting in the formation of distinct subpopulations [6–10]. These subpopulations can engulf dead cell debris and support tissue repair or secrete pro-inflammatory mediators that worsen tissue damage [8, 11–13]. Secondary damage contributes to the progressive deterioration observed in brain injury [14, 15], which involves a series of cellular and molecular processes, such as the release of cytokines, infiltration of inflammatory cells, increased permeability of the blood–brain barrier, oxidative stress, and cell death, among others [16].

Monocytes are MHC class II-expressing cells produced in the bone marrow [17] and give rise to classical, intermediate, and non-classical monocyte subsets [18, 19]. The non-classical subset has recently been implicated in mediating tissue damage after brain injury [3]. The phenotypic state of monocytes alters their ability to present antigens, produce pro-inflammatory cytokines, and express homing receptors. [20–23]. Monocytes must be metabolically flexible to retain their function in altered environments. For example, they rely on both oxidative phosphorylation and glycolysis for ATP production but shift their bioenergetic state during inflammation [24]. Recent evidence suggests that monocytes exhibit increased complexity and heterogeneity under pathological conditions. Severe inflammatory conditions can activate emergency monopoiesis, leading to the generation of functionally distinct subsets with alternative origins, including, for example, neutrophil-like monocytes, as revealed by single-cell transcriptomic analysis [20, 25, 26]. Emergency monopoiesis occurs when the bone marrow is stimulated to produce and release an increased number of monocytes into the bloodstream to increase monocyte frequency to help fight infection. This compensatory response plays a crucial role in resolving inflammation by migrating to the site of infection or tissue damage, where they contribute to the elimination of pathogens and the restoration of tissue homeostasis [27]. Whether emergency monopoiesis occurs following brain injury has yet to be described and the specific roles of altered monocyte subsets under these conditions in the pathophysiology of traumatic brain injury (TBI) remain ill-defined.

The present study aimed to evaluate the phenotypic characteristics of blood-enriched monocyte subsets at the single-cell level that emerge in response to brain injury with prior monocyte depletion using clodronate. We investigated whether altered monocyte subsets may be generated through emergency monopoiesis as a compensatory response to clodronate and their potential influence on injury outcomes. The monocytes that emerged into circulation after brain injury in mice pre-exposed to clodronate treatment could be distinguished by a featured gene expression profile and IPA pathway analysis that suggests a shift in bioenergetics, restriction of monocyte conversion, and the presence of a unique cluster not found in control cells, identified as neutrophil-like. Importantly, we show that the transfer of this monocyte assemblage to brain-injured mice was neuroprotective. Thus, our findings expand the understanding of monocyte subset influence on sterile brain injury and the key genes representing their heterogeneous classification and function.

## Results

### Monocyte depletion prior to controlled cortical impact (CCI) injury reduces tissue damage, blood–brain barrier (BBB) permeability and restores cortical blood perfusion

**Fig. 1:**
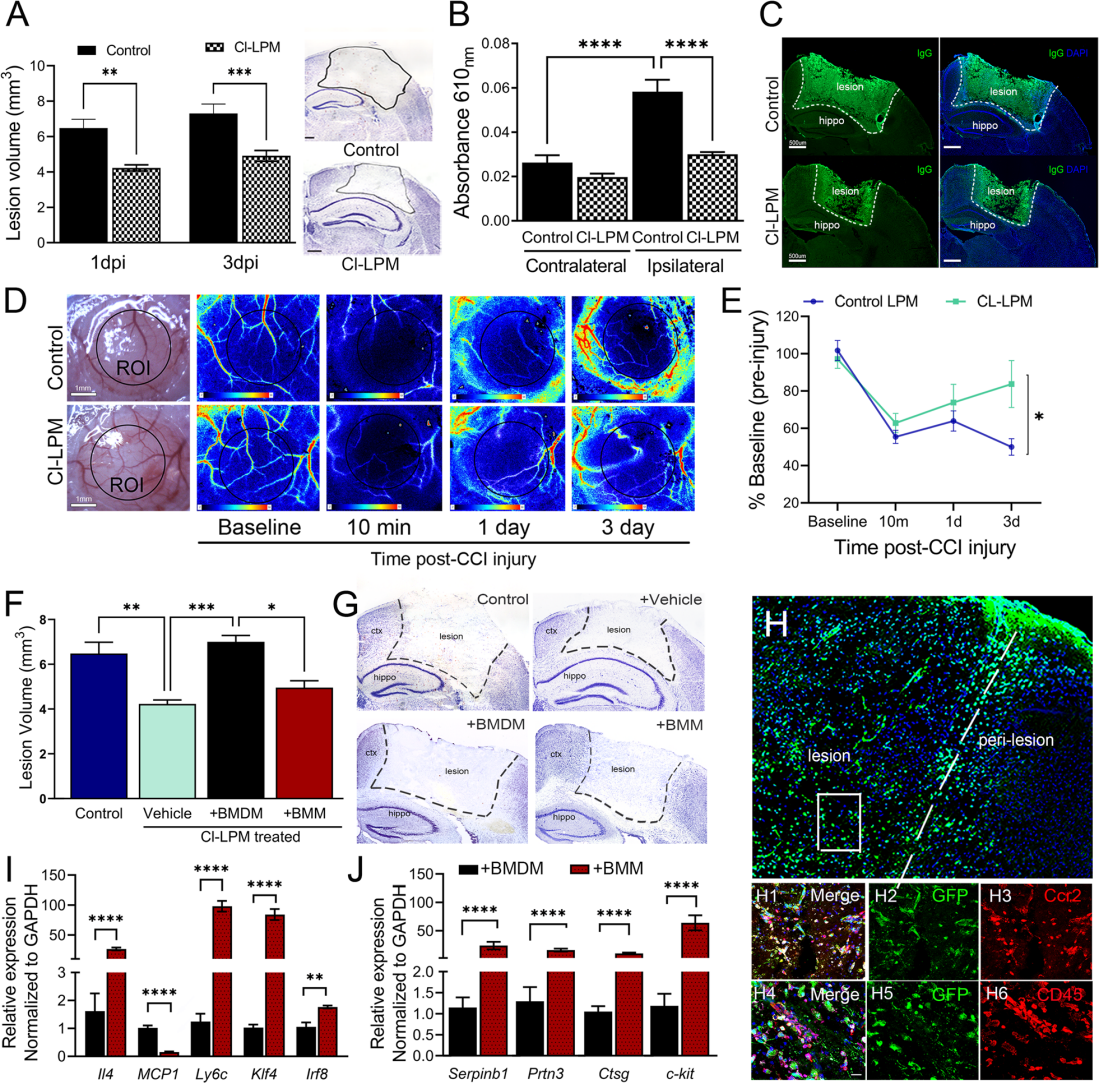
CCI-injured mice pre-treated with Cl-LPM show reduced lesion volume, maintained BBB integrity, and restored cortical blood perfusion. **A** Lesion volume at 1- and 3-dpi alongside representative Nissl images at 1dpi. **B** Evans blue absorbance (610 nm) analysis in the cortex at 1dpi. **C** Representative images for ipsilateral cortex of control and CL-LPM-treated mice stained with mouse anti-IgG (green) at 1dpi. **D** Representative gross images of baseline brain-meningeal surface after craniectomy and laser speckle contrast imaging. Scale bar = 1 mm. **E** Quantification of perfusion units (percent of baseline) following CCI injury. (*n* = 5–8 per group). *p < 0.05; **p < 0.01; ***P < 0.001; ****p < 0.0001. Two-way ANOVA with Bonferroni post hoc. Scale bar in *A* and *C* = 500 µm. **F** Lesion volume of control LPM or Cl-LPM treated mice, reconstituted with GFP^+^ BMMs or BMDMs at 1 dpi. **G** Representative Nissl-stained coronal sections. **H** Confocal image analysis showing GFP^+^ cells located in the damaged ipsilateral cortex, confirming co-labeling with monocyte-specific marker Ccr2 (**H1**–**H3**) or CD45 (**H4**–**H6**) Scale bar = 100um. **I**, **J** Relative mRNA expression in BMDMs vs. BMMs. *n* = 5–10 mice/group. **p < 0.01; ***p < 0.001, ****p < 0.0001. One-way ANOVA with Bonferroni post hoc (**B**, **F**); *t*-test (**A**, **I**, **J**); 2-way ANOVA repeated measures (**E**)

Clodronate is a small toxin encapsulated in liposome vesicles (LPMs) that induce targeted apoptosis of mononuclear phagocytes (monocytes, macrophages, dendritic cells) upon engulfment results in their selective depletion [28]. The effect on this population after brain injury and the subsets that emerge in brain injury alone or with prior exposure to clodronate were evaluated in this study. LPMs are accumulated in peripheral organs but not in the brain, following 2 sequential injections of Dil-labeled LPMs (3 days a part) (Additional file 1: Figure S1), which comport with previous studies using radiolabeled liposomes [29]. To investigate the role of mononuclear phagocytes in the sequelae of brain injury, clodronate liposomes (Cl-LPM) or control liposomes (control LPM) were i.v. injected at − 4 and − 1 days prior to unilateral, sham or CCI injury. We observed a significant reduction in lesion volume at 1- and 3 days post-injury (dpi) in Cl-LPM compared to control treated CCI-injured mice (Fig. 1A). Blood–brain barrier (BBB) disruption was also attenuated in the ipsilateral cortex by Evans blue and IgG deposition (Fig. 1B, C), and improved restoration of cerebral blood flow was observed in CL-LPM compared to control treated mice 3-dpi (Fig. 1D, E).

To confirm the clodronate effects were monocyte- and not dendritic-specific, we restored monocytes in the circulation of CL-LPM-depleted mice at the time of injury. GFP+ bone marrow monocytes (BMMs) (Additional file 2: Figure S2A) or ex vivo matured bone marrow-derived monocytes (BMDMs) were used to confirm cell-specific, as well as maturation effects. Restoration of monocyte/macrophages using BMDM attenuated tissue protection while BMM did not (Fig. 1F, G). We confirm the GFP^+^ cells infiltrate the damaged cortex (Fig. 1H) and co-label with monocyte marker Ccr2 (Fig. 1H1–H3; Additional file 2: Figure S2B) and immune marker CD45 (Fig. 1H5–H8). Interestingly, the mRNA expression profile between BMMs and BMDMs suggest that BMMs display higher *Ly6c* and immature markers *Klf4, Irf8, c-kit*, granule genes *Prtn3, Ctsg*, NE-elastase inhibitor *Serpinb1* and anti-inflammatory *Il4* expression compared to BMDMs which may explain the differential effects of BMMs on brain injury (Fig. 1I, J). These findings demonstrate that monocytes contribute to the histopathological sequela after brain injury, whose developmental state may be an important determinant in the course of tissue damage.

### Alterations in circulating myeloid populations in Cl-LPM CCI-injured mice

**Fig. 2:**
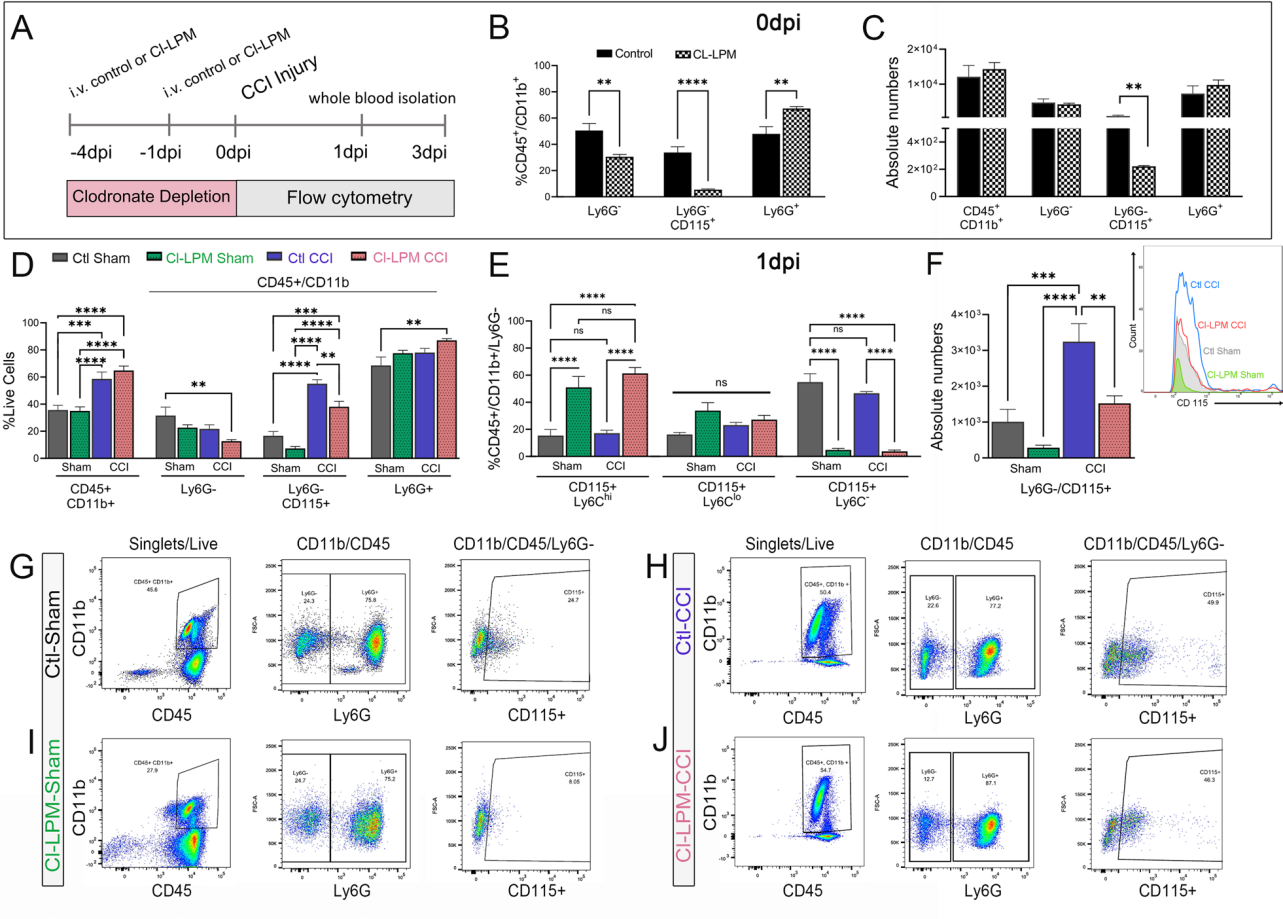
Monocyte population shift in blood following CCI injury and CL-LPM treatment. **A** Schematic representation of experimental timeline. **B**, **C** Flow cytometry analysis for the percentage (%) of CD45^+^/CD11b^+^ cell population that are Ly6G^−^, Ly6G^−^/CD115^+^, or Ly6G^+^ in CL-LPM or control-LPM treated naïve mice (0 dpi, **B**) as well as absolute numbers (**C**). **D**, **E** Flow cytometry analysis for the percentage (%) of myeloid population, including absolute numbers (**F**). **G**–**J** Flow cytometry gating strategy to select live/singlets, CD11b^+^/CD45^+^, and Ly6G^–^/CD115^+^ monocytes. *n* = 5 per group. *p < 0.05; **p < 0.01; ***p < 0.001; ****p < 0.00001. Student’s t-test (**B**, **C**); one-way ANOVA with Bonferroni post hoc (**D**–**F**)

We next assessed cell population shifts in circulation due to clodronate pre-treatment in sham and CCI-injured mice by evaluating the proportions of distinct cell populations. We collected whole blood and performed flow cytometry at 1dpi in mice that were pre-treated with control or Cl-LPM (Fig. 2A; schematic). Analysis of the proportion of cells in circulation was performed by quantifying the percentage (%) of CD45^+^/CD11b^+^ myeloid leukocytes, CD45^+^/CD11b^+^/Ly6G^−^, CD45^+^/CD11b^+^/Ly6G^+^, and monocytes (CD45^+^/CD11b^+^/Ly6G^−^/CD115^+^) in sham or CCI-injured mice exposed to either PBS LPMs or Cl-LPMs. Cl-LPM injections in non-injured naïve mice (0 dpi) show the percentage of Ly6G^−^ cells are reduced by ~ 40% compared to control LPM mice, and of these cells, the proportion of CD115+ monocytes are reduced by ~ 85% (5.5 ± 0.50%) relative to control (33.8 ± 4.3%), which corresponds to reduced absolute numbers (Fig. 2B and C). This confirms clodronate suppresses monocytes in circulation at the time mice are subjected to sham or CCI injury. Following control CCI injury, we observed a significant increase in the proportion and absolute numbers of monocytes (%CD45^+^/CD11b^+^/Ly6G^−^/CD115^+^) in circulation (Fig. 2D, F, H) by ~ threefold at 1 dpi compared to control sham levels (Fig. 2D, F, G). Surprisingly, we observed a ~ fivefold increase in Cl-LPM, CCI-injured mice (Fig. 2D, F, H) compared to Cl-LPM sham (Fig. 2D, F, G) at 1dpi. Ly6C^hi^ monocytes are the first subset to emigrate from the bone marrow in response to clodronate [30]. Consistent with this, we found an increase in Ly6G^−^/CD115^+^/Ly6C^hi^ monocytes in Cl-LPM sham and CCI-injured mice compared to control treatment groups at 1dpi (Fig. 2E). This shift coincides with a reduction in Ly6C^−^ cells. These findings suggest that clodronate pre-treatment may result in emergency monopoiesis, leading to an increased presence of Ly6C^hi^ monocyte subsets following TBI.

### ***Morphological analysis and mRNA characterization of whole blood immune cells show CD115***^+^***/Ly6C***^***hi***^*** neutrophil-like monocyte subset may emerge in Cl-LPM, CCI-injured mice***

**Fig. 3:**
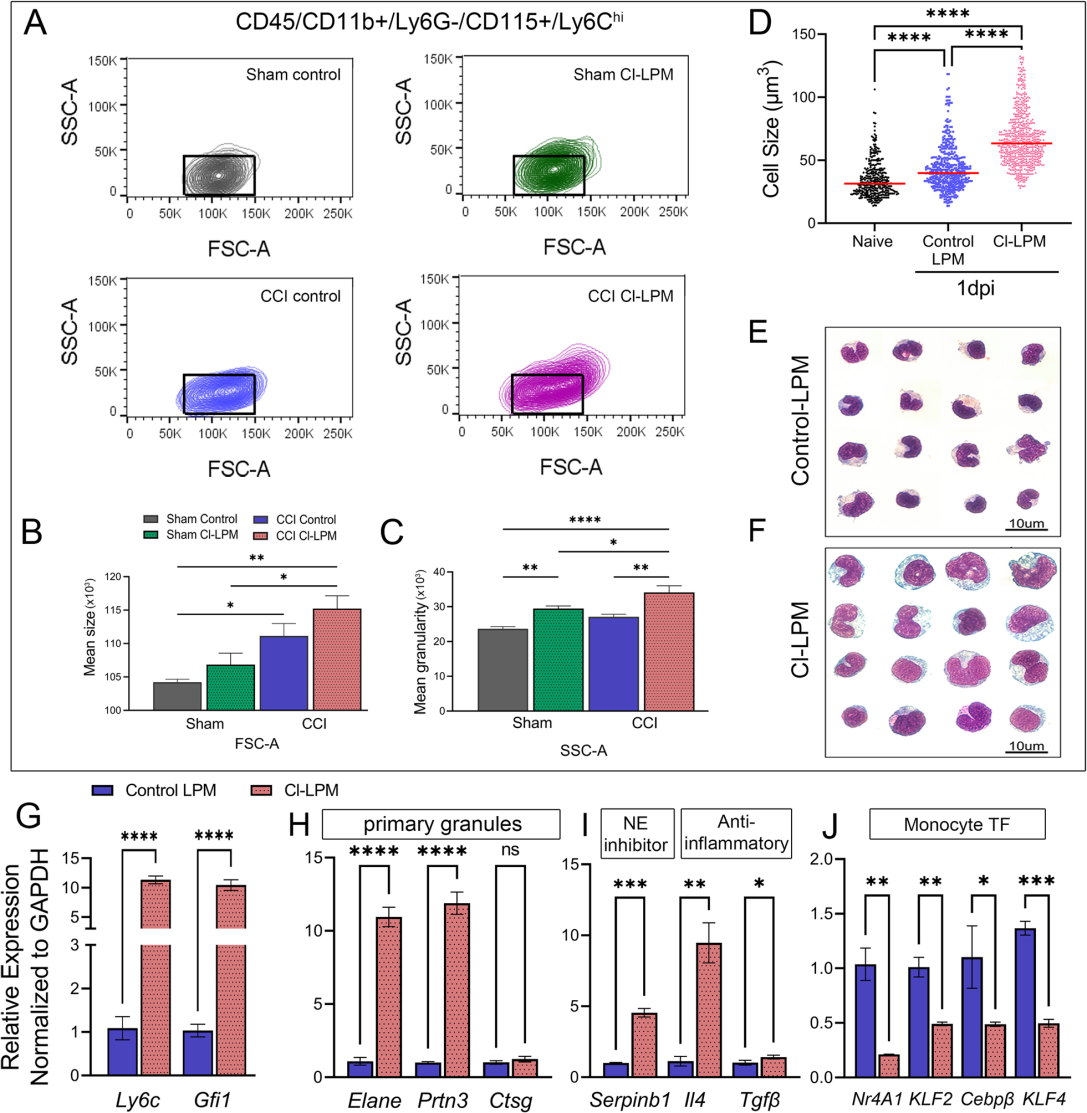
Distinctive morphology and gene expression signature in circulating CD115 + /Ly6C^hi^ monocytes of sham and CCI-injured mice pre-treated with control or Cl-LPM at 1dpi. **A** Representative contour plots of side (SSC-A) and forward (FSC-A) scattering of Ly6C^hi^ monocytes from sham and CCI-injured mice pre-treated with control or CL-LPM at 1 dpi. **B** The mean size (FSC-A) of Ly6C^hi^ monocytes is significantly increased in Cl-LPM or control LPM- treated CCI-injured mice compared to control sham cells. **C** The mean granularity (SSC-A) of Ly6C^hi^ monocytes is significantly increased in Cl-LPM-treated CCI-injured mice compared to other groups. **D** Cell size of whole blood monocytes from Cl-LPM treated mice at 1 dpi. **E**, **F** Representative images of differential quick cytology. **G**–**J** Relative mRNA expression *Ly6c* and neutrophil-like gene *Gfi1* (**G**), primary granules (**H**), anti-inflammatory (**I**), and monocytes TF (**J**) in circulating CL-LPM monocytes relative to control injured mice at 1 dpi. *n* = 5/group, *p < 0.05; **p < 0.01; ****p < 0.0001. One-way ANOVA with Bonferroni post hoc (**B**–**D**); *t*-test (**G**–**J**)

Following CD45^+^/CD11b^+^/Ly6G^−^/CD115^+^ gating, we further analyzed the Ly6C^hi^ monocyte subpopulation size and granularity by forward and side scatter (FSC-A and SSC-A, respectively). We observed an increase in FSC-A of Ly6C^hi^ monocytes from control (111,139 ± 1845.86) and Cl-LPM (115,221 ± 1923) CCI-injured mice compared to their respective sham groups (control, 104,229 ± 433.2; Cl-LPM, 106,836 ± 1717) (Fig. 3A, B) at 1dpi. However, the CD115^+^/Ly6C^hi^ monocytes from Cl-LPM, CCI-injured mice showed a significant shift toward larger mean granularity (34,132 ± 1885) compared to control CCI-injury (27,114 ± 746) (Fig. 3A, C) reminiscent of those with neutrophil-like characteristics [26]. To confirm the morphological changes detected by flow cytometry, we enriched for blood monocytes, evaluated cytology, and quantified their cell size at 1dpi (Fig. 3D–F). We detected a significant increase in monocyte size after injury, (naïve 34.9 um^3^ ± 14.3 vs 43.68 um^3^ ± 17.7) which was further increased in CL-LPM injured mice (67.4 um^3^ + 22). Analysis of mRNA transcript levels shows an increase in *Ly6c* (Fig. 3G), neutrophil-like transcription factors (TFs) *Gfi-1* and primary granules *Elane*, *Prtn3* and *Ctsg* (Fig. 3G and H) and a reduction in monocyte TFs *Nr4A1*, *Klf2, CepbB and Klf4* in whole blood monocytes isolated from Cl-LPM compared to control CCI-injured mice (Fig. 3J). Importantly, these cells show a significant increase in neutrophil elastase inhibitor, *Serpinb1* and anti-inflammatory genes *Il4* and *Tgfβ* (Fig 3I). Taken together, we find that the Ly6C^hi^ monocyte subset is characteristic of a neutrophil-like phenotype in Cl-LPM, CCI-injured mice.

### Phenotypic shift of infiltrating innate immune cells within the damaged cortex of Cl-LPM, CCI-injured mice

**Fig. 4:**
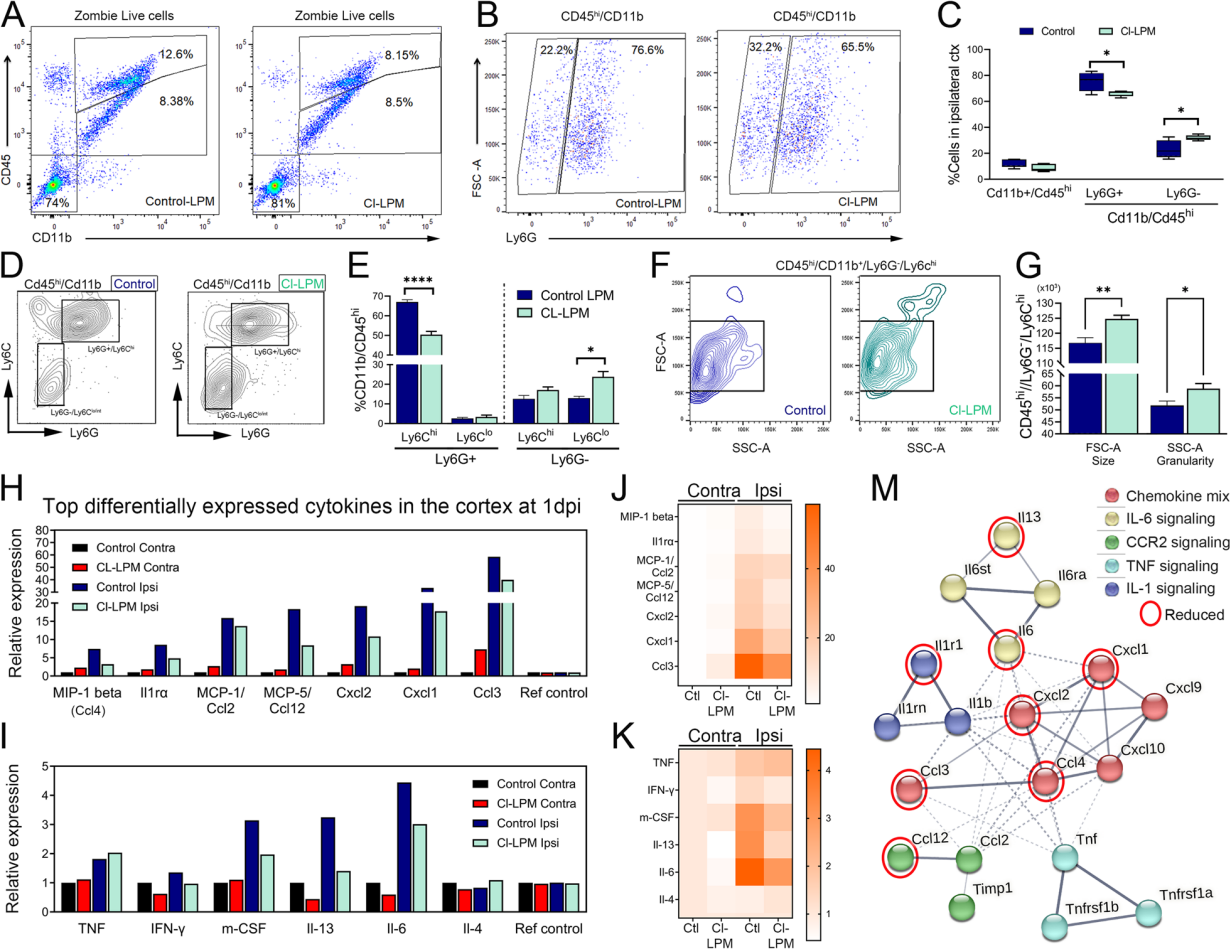
Phenotypic shift of infiltrating immune cells and reduced inflammatory cytokines in the cortex of Cl-LPM injured mice. **A**, **B** Flow cytometry gating for CD45^hi^/CD11b + cells and CD54^lo^/CD11b + microglia (**A**) followed by Ly6G^+^ selection of neutrophils and Ly6G^−^ myeloid (**B**). **C** Percentage of CD11b^+^/CD45^hi^ infiltrating immune cells, CD11b^+^/CD45^hi^/Ly6G^+^ neutrophils, and CD11b^+^/CD45^hi^/Ly6G-myeloid/macrophages in the ipsilateral cortex of Cl-LPM or control CCI-injured mice at 1 dpi. **D** Representative gating of Ly6C and Ly6G expressing cells from CD11b^+^/CD45^hi^ infiltrating immune cells in the damaged cortex. **E** The percentage of CD11b^+^/CD45^hi^/Ly6G^+^ and CD11b^+^/CD45^hi^/Ly6G^−^ cells co-labeled as Ly6C^hi^ or Ly6C^lo^ expression. **F** Representative FSC-A/SSC-A gating to evaluate size and granularity of Ly6C^hi^ cells in the injured cortex. **G** Quantification for the size and granularity of CD11b + /CD45^hi^/Ly6G-/Ly6C.^hi^ cell in the injured cortex at 1 dpi. **H**–K Cytokine Array protein quantification showing the top differentially expressed cytokines in the ipsilateral cortex at 1 dpi. **M** String analysis of the top differentially expressed cytokines. *n* = 5/group, *p < 0.05; **p < 0.01; ****p < 0.0001. *t*-test (**C**, **E**, **G**)

We evaluated the peripheral innate immune cell populations in the brain using flow cytometry at 1dpi by gating for CD45^hi^/CD11b^+^ cells to distinguish from CD45^lo^/CD11b^+^ resident microglia followed by Ly6G gating (Fig. 4A, B). First, we confirmed that CCR2, Ly6G, and Ly6C were not expressed by microglia using flow cytometry (Additional file 3: Figure S3D). Ly6G is expressed on neutrophils [31, 32] and CCR2 which is expressed exclusively on peripheral-derived monocytes/macrophages, but not resident microglia [33, 34] (Additional file 3: Figure S3C, D). Consistent with our histological findings of reduced Ly6G and increased Ccr2 in serial sections (Additional file 2: Figure S2A and B), dissociated ipsilateral cortical tissue from Cl-LPM, CCI-injured mice showed a significant reduction in the proportion of Ly6G^+^ neutrophils in CD11b^+^/CD45^hi^ gated cells compared to control treated CCI-injured mice 1dpi (Fig. 4C). This coincided with a significant percent increase in Ly6G^−^ myeloid cells (Fig. 4C) and reduced estimated total number of CD68^+^/ TMEM119^+^ in Cl-LPM compared to control mice at 3dpi (Additional file 3: Figure S3E). We further show that the Ly6G^+^/Ly6C^hi^ population is reduced, while the Ly6G^−^/Ly6C^lo^ was significantly increased in Cl-LPM compared to control CCI-injured mice (Fig. 4D, E). The SSC and FSC properties of CD45^hi^/CD11b/Ly6G^−^/Ly6C^hi^ cells in the damaged cortex at 1dpi showed increased size and granularity (Fig. 4F, G), similar to our findings in the circulation (Fig. 3).

To further characterize the cortical milieu at the protein level, we measured an array of 42 inflammatory cytokines in the injured cortex at 1dpi. We identify 15 cytokines with more than 2× upregulation in the ipsilateral cortex compared to contralateral. Interestingly, CL-LPM pre-treatment attenuated most differentially expressed inflammatory cytokines, including Cxcl2, Cxcl1 CCl3 m-CSF, IL13, and IL6 at the injured cortex (Fig. 4H–K). String analysis of these cytokines showed edges of association in 5 clusters encompassing key inflammatory players such as Ill, TNF, Ccr2, and Il6 in addition to an array of chemokines involved in monocyte, neutrophil, and NK chemotaxis (Fig. 4M). This data together suggests that alterations to circulating monocytes may directly influence the cytokine environment in the injured cortex as well as the peripheral and immune cell recruitment and phenotype.

### Single-cell sequencing of circulating monocytes shows distinct cluster profile in whole blood-enriched monocytes

**Fig. 5:**
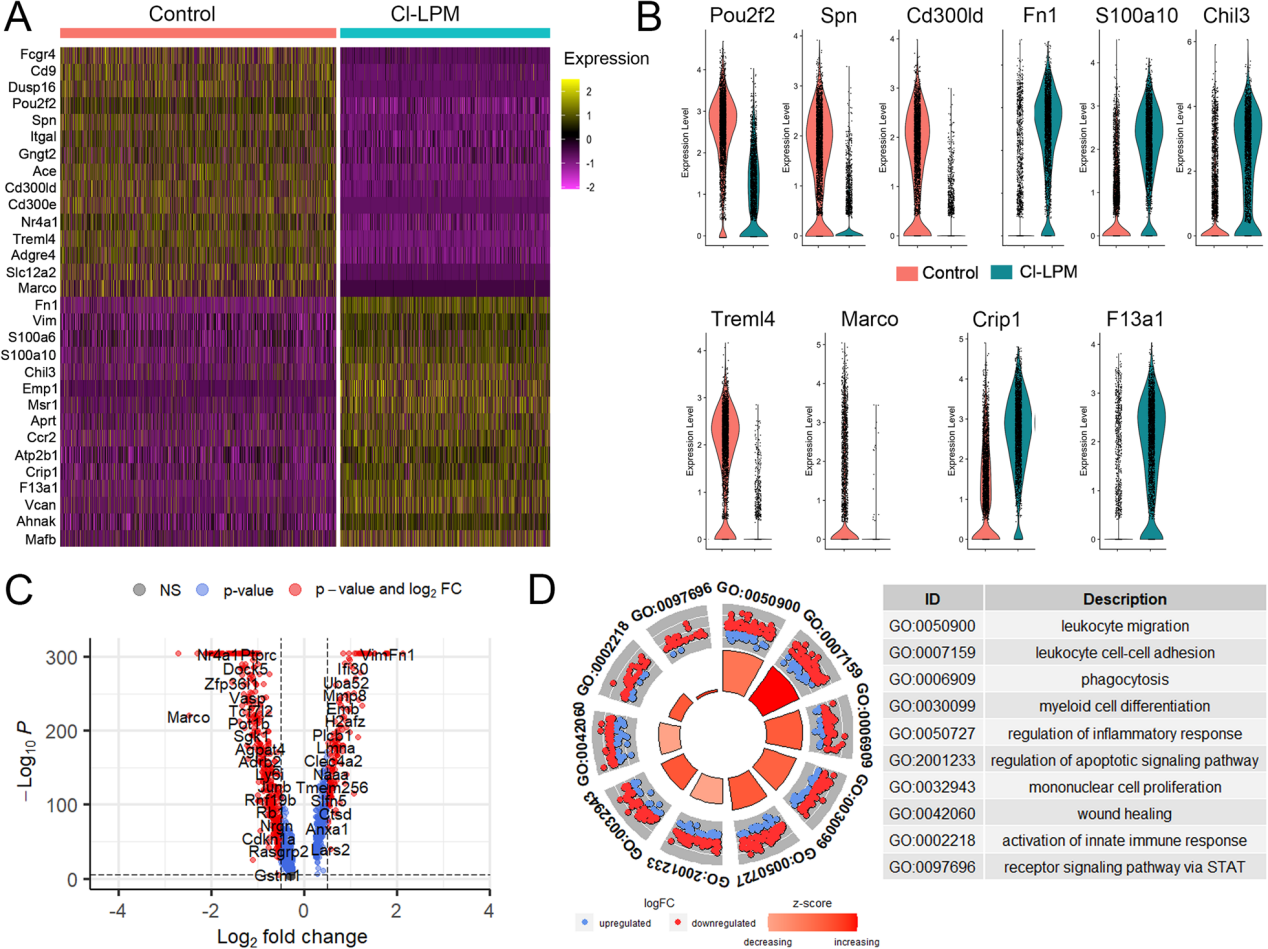
Single-cell RNA sequencing analysis shows distinctive transcriptomics of blood monocytes treated with Cl-LPM compared to control at 1 dpi. ScRNAseq was performed on monocytes isolated from blood of mice pre-treated with Cl-LPM or Control-LPM at 1 dpi. **A** Heatmap plotting of the top 15 up and down differentially expressed genes from CL-LPM compared to control-LPM pre-treated mice. **B** Violin plots of selected genes differentially expressed in CL-LPM monocytes vs control-LPM. **C** Enhanced volcano plot of DGEs. **D** GO plot shows top 10 relevant GO pathways related to all significant up and down regulated genes in CL-LPM relative to control monocytes

To examine the heterogeneity of monocytes in circulation after CCI injury, scRNA-seq was used to identify cellular signatures in control to pre-depleted mice. A total of 4663 cells from Control and 4201 for Cl-LPM were included for analysis. On average, 7055 reads per cell and 2464 genes per cell were sequenced, detecting more than 17.600 genes in total. Based on the sequencing results, Seurat automated cell type analysis showed an expression profile characteristic of monocytes, confirming the successful enrichment of magnetic isolation of monocytes (Additional file 4: Figure S4). We first compared the differential gene expression profile of all monocytes from Cl-LPM pre-treated mice relative to control-LPM monocytes at 1dpi and observed significant differences in gene expression (Fig. 5A, C). The top 15 differentially expressed genes are displayed on the heatmap and volcano plot shows 515 downregulated and 1070 upregulated genes, demonstrating clear transcriptomic differences. Cl-LPM monocytes show reduced expression of *Pou2f2, Spn, Cd300lde, Treml4, Marco* which mainly represent alternative monocyte genes and pattern recognition receptors (Marco) but increased *Fn1, S100a10, Chil3, Crip1, F13a1* that are involved in immunomodulation, granulocyte-like transcriptional features, and cell adhesion (Fig. 5B). The top 10 most significant biological processes (BP) terms, that represent all genes with adjusted *p*-value < 0.05, indicate that the DGE pattern relates to changes in monocyte adhesion, migration, differentiation, and regulation of the inflammatory response (Fig. 5D).

**Fig. 6:**
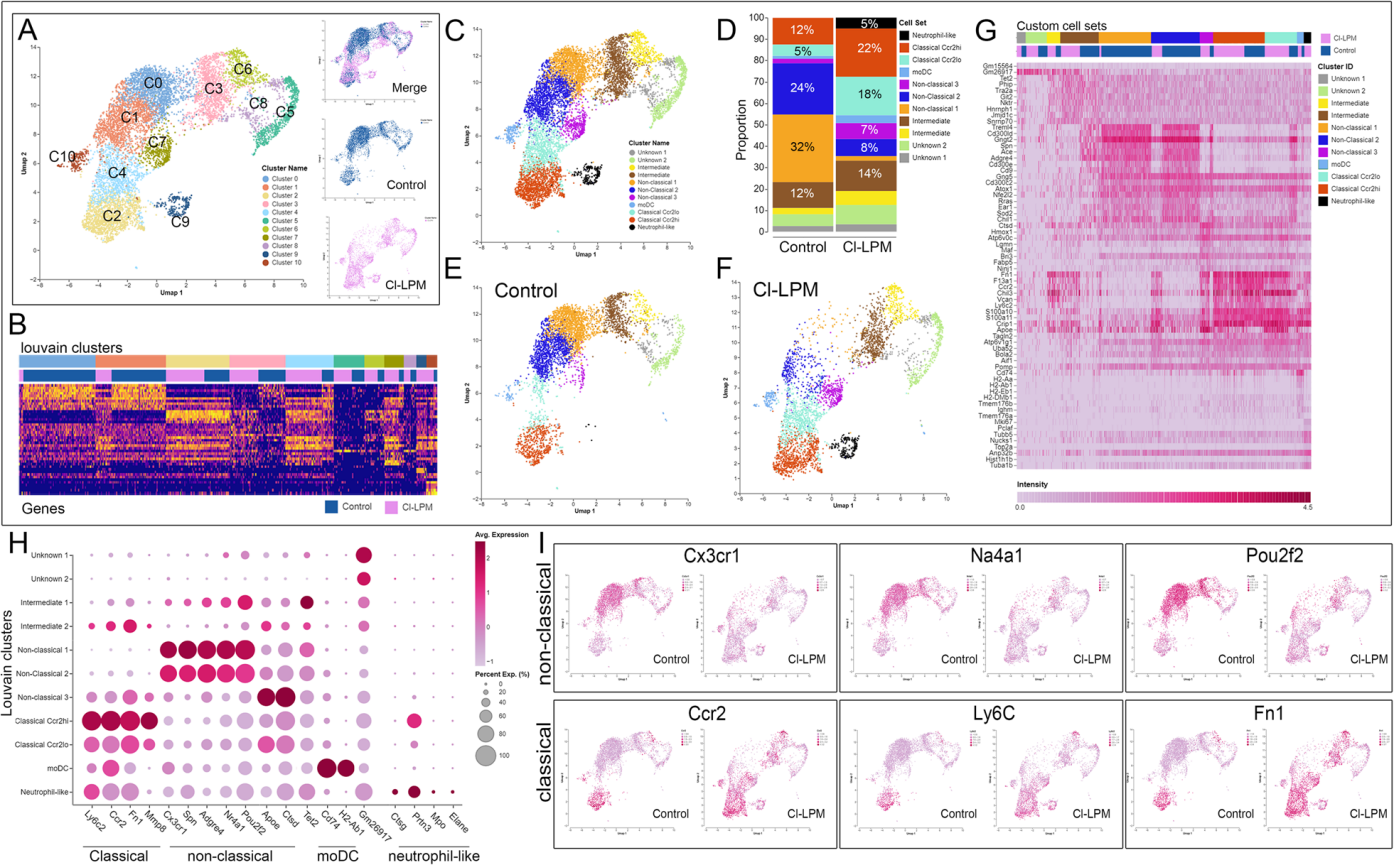
scRNAseq analysis displays altered monocyte clustering for blood monocytes isolated from Cl-LPM and control mice at 1 dpi. **A** Monocytes from control and Cl-LPM treated mice are clustered based on RNA gene expression in a Uniform Manifold Approximation and Projection (UMAP) plot. **B** Heatmap distinguishing each of the 11 clusters between samples. **C** UMAP following custom cell annotation and the percentage of cell types in each sample (**D**). **E**, **F** UMAP of control and Cl-LPM, respectively. **G** Heatmaps of top 10 up DEG’s DGE (Y axis) per cluster (X axis) for control and Cl-LPM samples showing distinctive gene expression profiles. **H** Dot plot of top genes expressed by annotated cell types. **I** Feature maps highlighting spatial expression across clusters of *Cx3cr1, Na4a1, Pou2f2, Ccr2, Ly6c, Fn1* in control and Cl-LPM cells

The enriched monocytes were clustered using Seurat method on Cellenics, an open-source, cloud-based analytic tool for scRNA-seq data (Biomage). Monocytes enriched from the whole blood displayed 11 clusters (C0–C10), and the patterns of these clusters differed between Control and Cl-LPM groups (Fig. 6A, inset, 6B). Subsets were then classified as classical, intermediate, and non-classical (alternative) and monocyte-derived dendritic cells (MoDC) based on custom gene annotation (Fig. 6C). Notably, control monocytes showed a distinct aggregation of C0, C1, C2 while Cl-LPM consisted of C2, C4, C7, C9. Based on the gene set annotation, the cluster profile indicates that Cl-LPM monocyte phenotypic state is altered from non-classical to classical. Analysis of the proportion of monocytes revealed that 56% of the control were non-classical type 1 and type 2 (Fig. 6D, E), while 52% of Cl-LPM were Classical Ccr2^hi^, Classical Ccr2^lo^, a non-classical subtype 3 and neutrophil-like (Fig. 6D, F). Heatmap representation of the top 10 DGEs (Fig. 6G) show distinct differences between monocyte clusters and samples (Fig. 6G), with key genes showing highest expression in each annotated cell types (Fig. 6H) including *Ly6c2, Ccr2, Fn1* and *Mmp8* (classical); *Cx3cr1, Spn, Adgre4, Nr4a1, Pou2f2* (non-classical 1 and 2); *Apoe, Ctsd* (non-classical 3); *Cd74, H2-Ab1* (moDC); *Ctsg, Prtn3, Mpo* and *Elane* (neutrophil-like). Feature UMAPs further highlight the cell specific  location of these genes in the different clusters confirming their enriched expression (Fig. 6I). A summary of these changes (Table 1) detail the DGE for granule function, monocyte subset identity and inflammation-associated pathways. These findings demonstrate that circulating monocytes that emerge after CCI injury in mice with prior exposure to clodronate, have an altered phenotypic state that include the presence of a distinct neutrophil-like, and a Ccr2^lo^ classical subpopulation not observed in control.

**Table 1:** Raw expression values from whole blood enriched monocytes

Genes	(1) pct Control^*^	(2) pct CL-LPM^*^	Protein name	Function
Monocyte (all clusters)				
Ly6c1	ns	ns	Lymphocyte antigen 6C	Involved in acetylcholine receptor signaling pathway
Ly6c2	0.165	0.668	Lymphocyte antigen 6C	Involved in acetylcholine receptor signaling pathway
Ccr2	0.217	0.81	CC motif chemokine receptor 2	Migration into tissue
Non-classical monocyte (all clusters)				
Cx3cr1	0.74	0.467	Growth Factor Independent 1 Transcriptional Repressor	Non-classical monocytes
Spn	0.762	0.26	Sialophorin	Alternative macrophage marker
Cebpb	0.959	0.902	CCAAT Enhancer Binding Protein Beta	TF for conversion of classical *monocytes* into non-classical *monocytes*
Klf2	0.86	0.511	KLF transcription factor 2	TF for conversion of classical *monocytes* into non-classical *monocytes; pro-inflammation*
Pou2f2	0.948	0.64	POU Class 2 Homeobox 2	Marker non-classical monocytes
Nr4a1	0.873	0.44	Nuclear receptor subfamily 4 group A	TF for conversion of classical *monocytes* into non-classical *monocytes*
Adgre4	0.834	0.378	Adhesion G protein-coupled receptor E4	Novel marker for non-classical
Azurophilic granules (C9)				
Elane	0.39	14.02	Neutrophil elastase	Neutrophil granule serine protease, elastase
Prtn3	19.62	60.28	Proteinase 3	Serine protease enzyme in neutrophil granules
Ctsg	1.86	27.1	Cathepsin G	Serine protease enzyme in neutrophil granules
MPO	0.45	14.49	Myeloperoxidase	
Neutrophil-specific granules (all clusters)				
Mmp8	0.114	0.517	Matrix metalloproteinase-8	Neutrophil collagenase
Lyz2	0.982	0.996	Lysozyme	Neutrophil lineage gene
Lcn2	0.098	0.34	Lipocalin	Neutrophil gelatinase-associated lipocalin (NGAL)
Inflammation-associated (all clusters)				
Nfkbia	0.882	0.644	Nuclear factor-kappa-B-inhibitor alpha	Produces alpha subunit of Ikk protein complex
Nfkbiz	0.778	0.383	IkappaB-zeta	Transcriptional regulator for NF-kappaB
Il1b	0.523	0.054	Interleukin-1 beta	Pro-inflammatory immune response
Tgfbi	0.583	0.905	Transforming growth factor beta-induced	Immunosuppressive
Marco	0.326	0.007	Macrophage receptor with collagenous	Pattern recognition receptor; recognition and clearance of pathogens

^*^Single-cell data (pct.), < 0.05 adj *p*-value

### Analysis of single-cell sequencing reveals distinct gene profile, ontology, and IPA pathways

**Fig. 7:**
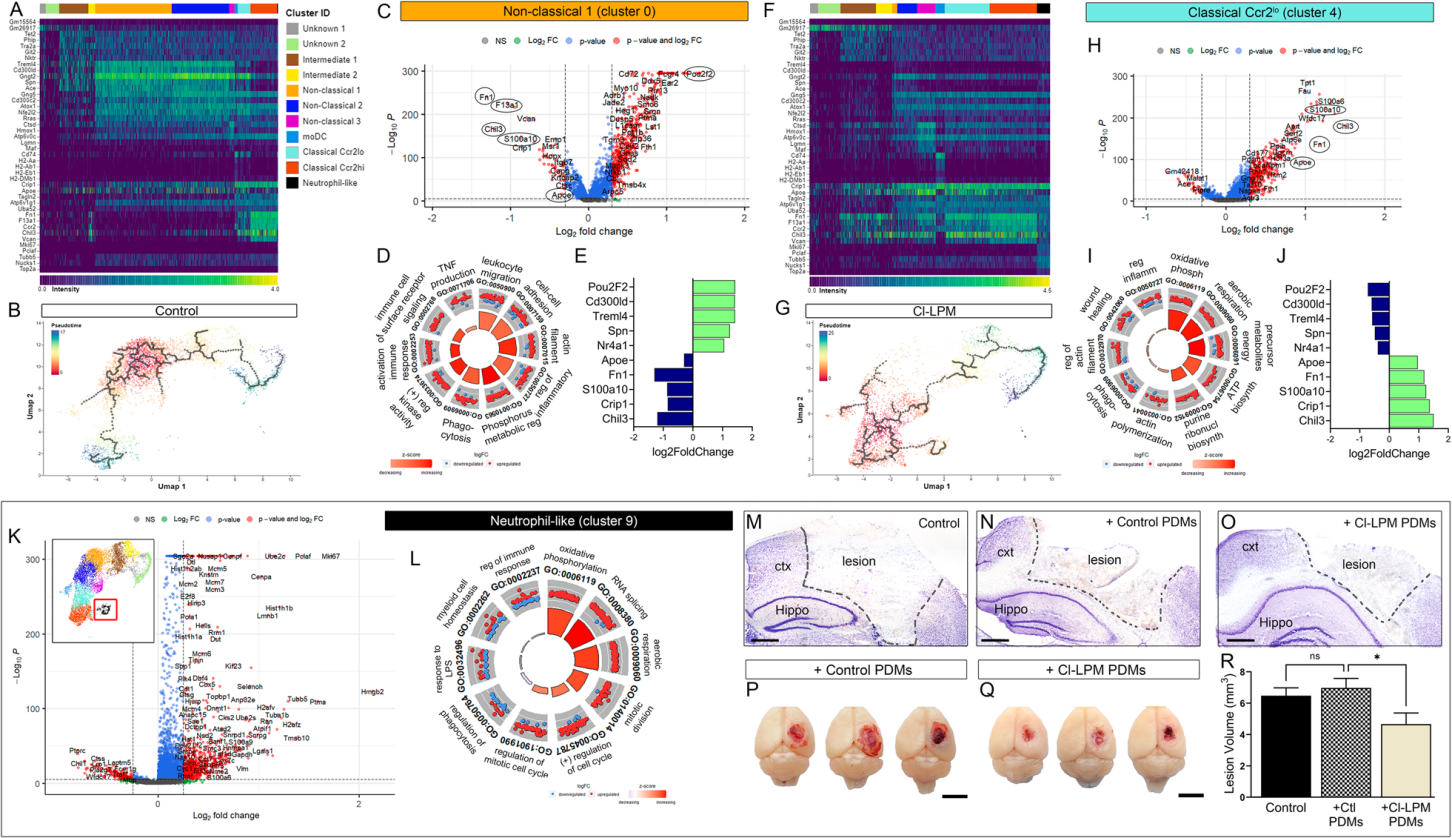
Top DGEs and Gene Ontology using scRNAseq cluster analysis of control and Cl-LPM monocytes. **A** Heatmap of top 10 genes in each annotated cell cluster in control monocytes. **B** Cell trajectory analysis using pseudotime shows a hub in Cluster (C) 0. **C** Volcano plot of DGEs in C0, non-classical. **D** GO analysis shows migration, cell–cell adhesion, actin assembly, regulation of inflammatory response and TNF production as top biological processes in C0. **E** Top 5 UP and down regulated genes in C0 that show inverse expression compared to C4. **F** Heatmap of top 10 genes in each annotated cell cluster in Cl-LPM monocytes. **G** Cell trajectory analysis using pseudotime. **H** Volcano plot of DGEs in C0, non-classical. **I** GO analysis shows oxidative phosphorylation, aerobic respiration, etc., as top biological processes in C0. **J** Top 5 UP and down regulated genes in C0. **K** Analysis of top DGE in neutrophil-like monocytes, C9. **L** GO analysis for C0. **M**–**O** Transfer of peripheral-derived monocytes (PDMs) by i.v. injection immediately following CCI injury. **P**, **Q** Gross images of injured brain at 1dpi shows tissue protection in mice receiving Cl-LPM PDMs (**R**). *n* = 5–8 *p < 0.05; one-way ANOVA, Bonferroni post hoc. Scale = 500 um in **M**–O and 0.5 cm in **P** and **Q**

Additional investigation into the genes expressed by distinct monocyte clusters showed C0, non-classical 1 predominant in control monocytes, displayed a notable profile of differentially expressed genes (DEGs) compared to all other clusters (Fig. 7A, C). This includes higher expression of non-classical monocyte markers *Spn*, *Pou2f2,* and transcription factor *Na4a1*, which are involved in converting from classical to non-classical phenotype (Fig. 7E). Gene Ontology (GO) analysis, 1.2FC and < 0.05 adj *p*-value, indicate the biological processes (BP) most highly regulated include TNF production, migration, cell–cell adhesion, and activation of immune response (Fig. 7D). Conversely, C4 classical Ccr2^lo^, mainly present in Cl-LPM monocytes, showed increased expression of *Chil3, Crip1, S100a100, Fn1* and *Apoe* (Fig. 7F, H, J). Interestingly, we observed that C0 and C4 showed an inverse expression profile for these top genes (Fig. 7E vs J). Moreover, GO analysis revealed oxidative phosphorylation, aerobic respiration and ATP biosynthesis as top pathways regulated by C4 DEGs (Fig. 7I). Further, C9, neutrophil-like subset DEGs include 728 upregulated genes including *Hmgb2, Mki67, Ptma, Pclaf, Tubb5* (Fig. 7K). Similar to C4, C9 also exhibits oxidative phosphorylation, aerobic respiration as top BP, as well as mitotic division and cell cycle regulation indicating these cells may have higher proliferative capacity (Fig. 7L). Pseudotime analysis for C0 and C4, demonstrated a center hub in C0 and C4 with distinct trajectories, indicating potential alterations in their differentiation program (Fig. 7B and G, respectively). Interestingly, the trajectories of pseudotime analysis in Cl-LPM suggest that C9 (neutrophil-like monocytes) differentiate from classical monocytes. (Fig. 7G). Further, C9, neutrophil-like (NLMs) subset present in Cl-LPM, DEGs include 728 upregulated genes including Hmgb2, Mki67, Ptma, Pclaf, Tubb5 (Fig. 7K). Similar to C4, C9 also exhibits oxidative phosphorylation, aerobic respiration as top BP, as well as mitotic division and cell cycle regulation indicating these cells may have acquired a proliferative phenotype (Fig. 7L).

To examine the direct functional role of Cl-LPM monocyte subsets on tissue damage, we isolated Cl-LPM or control monocytes from CCI-injured whole blood and i.v. injected 150k cells into recipient mice immediately following cortical impact. While injection of control peripheral-derived monocytes (PDMs) had no effect on lesion volume at 1dpi (Fig. 7N, P, R) compared to vehicle-injected mice (Fig. 7M, R), we observed that Cl-LPM PDMs resulted in reduced lesion volume (Fig. 7O, Q, R). This data suggests that the supplementation of monocytes generated under emergency hematopoiesis have neuroprotective properties in the brain following injury.

**Fig. 8:**
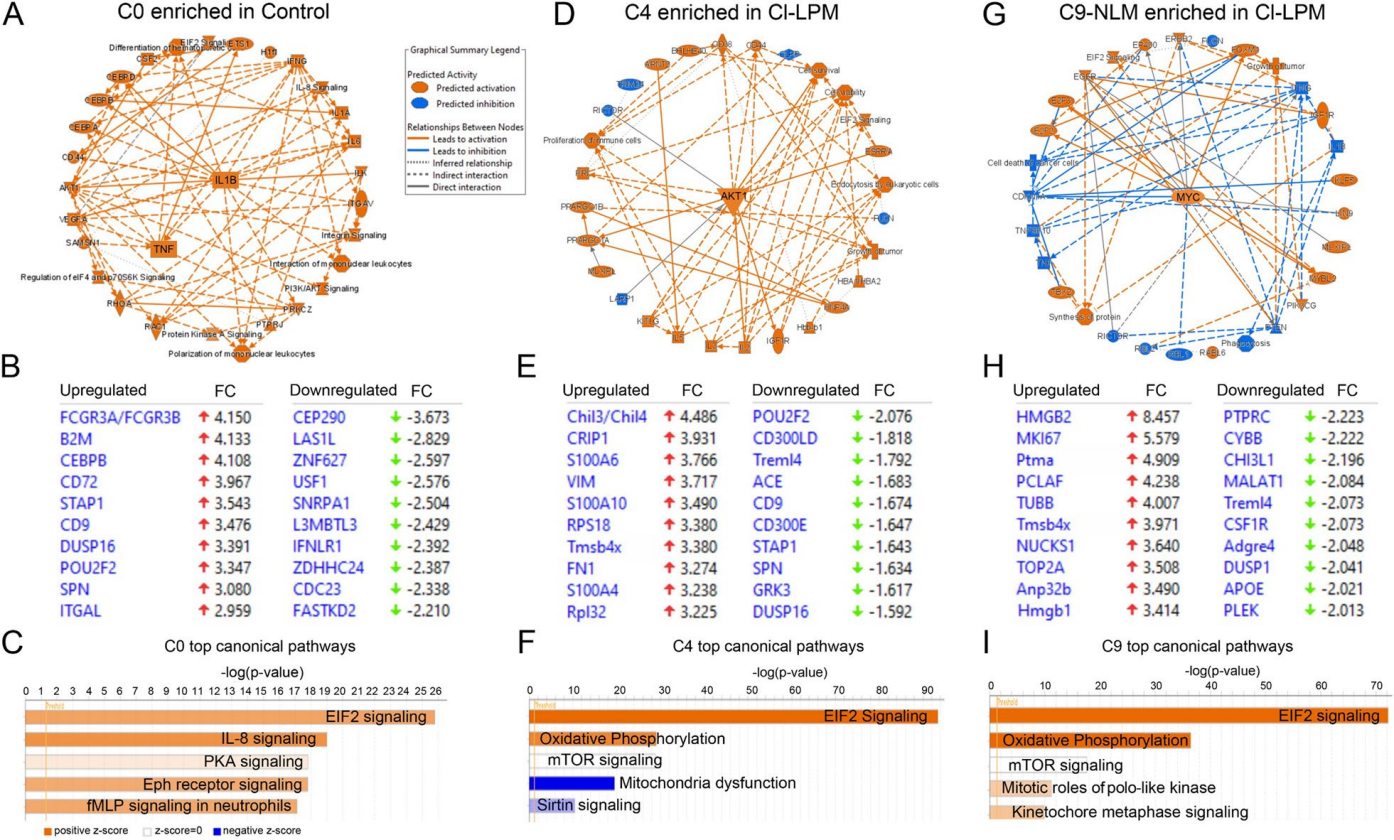
IPA analysis of C0, C4 and C9. **A**, **D**, **G** Graphical summary of top signaling predicted to be activated (orange) or inhibited (blue). **B**, **E**, **H** Top analysis ready molecules. **C**, **F**, **I** Horizontal bar of top canonical pathways identified for each monocyte cluster base don −log(*p*-value). Orange = predicted to be activated; blue = predicted to be inhibited

To gain greater insight into the underlying mechanisms and regulatory pathways driving the observed changes in genes expression (Fig. 8B, E, H; top genes), we performed IPA analysis, which predicted activation of TNF and IL1B in C0, AKT1 in C4 and MYC in C9 (Fig. 8A, D, G; respectively). The activation of TNF and IL1B in C0 suggests an inflammatory response unique to the enhancement of Il-8, PKA, Eph and fMLP canonical signaling pathways (Fig. 8C). The common predicted EIF2 pathway across all clusters indicates a general cellular stress response (Fig. 8C, F, I). Moreover, the enrichment of oxidative phosphorylation in C4 and C9 (Fig. 8F, I) is consistent with GO analysis and highlights the potential metabolic adaptations in these clusters. Mitotic PLK and kinetochore metaphase pathways suggest additional cell cycle regulation in C9. These findings provide critical insights into the intricate regulatory networks that govern gene expression in monocytes subsets.

## Discussion

Brain injury causes rapid recruitment of innate immune cells to areas of tissue damage, where monocytes represent a major constituent of this response. Clodronate liposomes (CL-LPM) have been widely employed for monocyte depletion studies to investigate in vivo functionality. The current study revealed that brain injury in mice with prior exposure to clodronate, yielded substantial acute histopathological and functional benefits. Analysis of monocytes in circulation, revealed that CCI injury significantly elevated their levels in mice with pre-injury exposure to clodronate, compared to clodronate alone, and resulted in the presence of a neutrophil-like subset. This robust response may be indicative of emergency monopoiesis [23, 26]. Flow cytometry showed that a greater proportion of Cl-LPM, CCI-injured CD115^+^ monocytes were Ly6C^hi^, which were larger, more granular and expressed neutrophil-like gene *Gfi1* and granule genes *Elane, Prtn3* and *Ctsg*. This phenotype coincided with reduced proportions of CD45^hi^/Ly6G^+^ neutrophils and greater CD45^hi^/Ly6G^−^ myeloid/macrophage cells in the damaged cortex that were larger and granular. Moreover, the injured cortical milieu of Cl-LPM, CCI-injured mice harbored reduced expression of Ccl3, Cxcl1, Cxcl2, Ccl12 and Il6 pro-inflammatory cytokines. Importantly, scRNAseq of enriched whole blood monocytes confirmed the unique presence of a neutrophil-like subset only in circulation of Cl-LPM CCI-injured mice. However, the predominate subset in Cl-LPM, CCI-injured mice were classical (Ccr2^hi^ and Ccr2^lo^) rather than non-classical, which have been implicated in tissue damage in this model [3]. Passive transfer of this emergent population conferred neuroprotection demonstrating their direct role in modulating tissue health after injury. Overall, findings from this study offer new insight into the regulatory role of monocyte subsets in the brain.

Circulating monocytes are mononuclear phagocytes that differentiate upon entrance into injured tissue [2, 3, 35] and their infiltration and differentiation has been described in the brain following trauma [2, 36]. Inflammatory monocytes traffic to the brain and spinal cord in human and rodent models [37–40]. Their release from the bone marrow has been shown to contribute to their recruitment, however, the subcapsular red pulp of the spleen represents an alternative source of monocytes [41] and splenectomy was shown to decrease macrophages in the spinal cord after injury which correlated with improved motor recovery [41]. Interestingly, we find that *Treml4* is among the highest genes expressed in C0, non-classical subtype in control monocytes, and is expressed at high levels in red pulp of the spleen [42]. Given that Cl-LPM treatment eliminates monocyte/macrophages in the red pulp, resulting in reduced spleen weight (Additional file 5: Figure S5A–E), this suggests the reduction in availability of splenic monocytes could shift the emergent source of circulating monocytes from spleen to bone marrow. Moreover, under resting conditions, primary splenic macrophages produce higher levels of pro-inflammatory cytokines than those from the bone marrow or peritoneum [43]. Further, studies show that monocytes that reappear in circulation after Cl-LPM treatment are exclusively Ly6C^hi^ bone marrow monocytes [44]. We cannot rule out the possibility that Cl-LPM alone in naïve mice may generate the same phenotypic changes in circulating monocytes. Analysis of our flow scatter data, however, suggests that CCI injury may be needed to enhance these effects and contribute to the release of an immunoregulatory population.

Prior studies in mice confirm the neuroprotective effects of pre-injury depletion with clodronate and also detail the role of non-classical monocytes in tissue damage [3], while studies in rats indicate pre-depletion increases BBB disruption [45] and opposing effects have been observed in stroke outcome [46, 47]. These divergent findings may be due to species, model or injury severity differences, or investigative tools which warrant further investigation. We are the first to show this hyperacute compensatory mechanism using outbred CD1 mice as early as 24 h after brain injury. Others have used altered mouse strains [3], confirmed clodronate effects only prior to injury [48] or used spleen and brain for outcome measures [47, 49]. Collectively, however, these studies highlight the key importance of monocytes in pre-clinical models of neurological impairments.

Recent findings reveal that murine bone marrow granulocyte-monocyte progenitor (GMP) cells independently produce a distinct subset of Ly6C^hi^ neutrophil-like monocytes (NLMs), which in humans are classified as CXCR1^+^CD14^+^CD16^−^ subset [26, 50]. GMPs can generate NLMs following LPS but not CpG exposure, which is considered a distinct pathway in emergency monopoiesis in response to pathogens [26]. Interestingly, clodronate has been shown to cause bacterial translocation in whole blood [51]. NLM generation has also been shown following cyclophosphamide treatment, whose response is regulated by DNMT1 and microbial translocation [52]. Indeed, we have observed the presence of bacterial cell wall fragments (LPS) in the serum of Cl-LPM, CCI-injured mice but not in CCI alone (Additional file 5: Figure S5H), suggesting a possible role for GMP-mediated production of NLMs by gut-derived microbe-associated molecular patterns (MAMPs) in Cl-LPM mice. Whether this potential response could be further altered by brain injury remains to be investigated.

Analysis of scRNAseq clusters reveal that the collective Cl-LPM monocyte population have higher *Ly6c2* and *Chil3/Ym1* expression. Ly6C^hi^/Ym1^+^ alternatively activated monocytes contribute to the resolution of inflammation and tissue repair caused by systemic inflammation [53]. Furthermore, we find that the factors controlling conversion from Ly6c^hi^ to Ly6c^lo^ (*Klf2, Cebpb, Nr4a1, Pou2f2, and Adgre4*) and non-classical identity (*Cx3cr1, Spn*) were reduced in Cl-LPM monocytes suggesting the monocytes may remain Ly6C^hi^, which could contribute to tissue protection. In addition, transforming growth factor beta induced (*Tgfbi*) is increased in Cl-LPM monocytes, while *Nfkbia, Nfkbiz, Il1b* and *Marco* are reduced in scRNAseq and *Il4* is increased by qPCR in Cl-LPM cortex (Additional file 3: Figure S3F), which may contribute to an immunosuppressive environment [54]. Importantly, we observed that the predominate biological process and canonical IPA pathway identified for C4 was oxidative phosphorylation. Oxidative phosphorylation in monocytes is important for resolving inflammation by providing ATP for cellular functions, supporting phagocytosis and efferocytosis, maintaining redox balance, facilitating metabolic reprogramming, and enabling the production of specialized pro-resolving lipid mediators [55, 56]. At the peak of inflammation, immune cells preferentially use glycolysis as a source of energy. In contrast, during the resolution phase, they rely mainly on OXPHOS metabolism, acquiring a pro‐resolving phenotype [57]. Efficient oxidative phosphorylation ensures the proper functioning of monocytes during the resolution phase of inflammation [58]. Thus, in recent years it has become evident that different metabolic routes determine the fate of immune cells and impact the inflammatory response. Future studies are needed to validate the source of monocyte subsets and how key metabolic adaptations directly support their identity and function.

In conclusion, under defined conditions a functionally and molecularly distinct subset of circulating monocytes emerge following brain injury. This work emphasizes the necessity for further investigation into the origin and phenotypic role of monocytes in regulating TBI-induced neurological sequelae.

## Methods

### Animals

All animals were housed in an AAALAC accredited, virus/antigen-free facility with a 12 h light–dark cycle. Mice used were on the CD1 IGS outbred strain (Charles River, Durham, NC) and were provided food and water ad libitum. Procedures were performed on male mice at 8–10 weeks of age. All experiments were conducted in accordance with the NIH Guide for the Care and Use of Laboratory.

### Systemic depletion of monocytes and Dil liposome experiments

Mice were treated with 200 µl of clodronate liposomes (Cl-LPM) (Liposoma, Netherlands) or control PBS liposomes (Control LPM) or Dil-labeled liposomes (Dil-LPM) via tail vein injection at 4- and 1-day prior to CCI or sham injury. For quantification of Dil-LPM uptake, blood smears were prepared, and slides were evaluated by fluorescent and brightfield microscopy to quantify % of Dil-labeled WBCs.

### Controlled cortical impact (CCI)

Surgeries were performed as previously described [59–62]. Briefly, mice were anesthetized with ketamine 100 mg/kg and xylazine 10 mg/kg. Buprenorphine SR (0.5 mg/kg) was also administered SQ prior to surgery. A *Φ* = 4 mm craniotomy was made over the right parietal bone (− 2.5 mm A/P and 2.0 mm lateral from bregma) and cortical injury was induced using a programmable electronic controlled cortical impactor (*Φ* = 3 mm round tip) connected to an eCCI-6.3 device (Custom Design & Fabrication, LLC) at a velocity of 5.0 m/s, depth of 2.0 mm, and 150 ms dwell time.

### Laser speckle contrast imaging (LSCI)

LSCI was acquired using a Laser Speckle Imaging System III (RWD Life Science, Dover, DE, USA). Scanning occurred over the right parietal cortex with 3 × zoom. Each mouse was recorded four times every 10 s over a region of interest of 2.5 m diameter before injury or baseline, then at 10 min, 1- and 3-dpi. The average of each recording was graphed as relative to the mean baseline average. Laser speckle contrast images and bright field images were taken at every recording.

### Bone marrow-derived monocyte/macrophage (BMDM) cell culture

Bone marrow-derived monocyte/macrophages were isolated from 8- to 10-week-old GFP reporter mice on CD-1 background as described [63]. Briefly, bone marrow cells were filtered through a 40-μm strainer, red blood cells were lysed using ACK lysing buffer (ThermoFisher, Gaithersburg, MD), and were cultured at 1 × 10^6^ cells/ml in 10 cm dishes containing DMEM (#D6546 Sigma St Louis MO) medium supplemented with 10% fetal bovine serum, 2 mM l-glutamine, 1% penicillin/streptomycin and 10 ng/ml M-CSF. Media changes occurred on days 2 and 4, then on day 5 of culture, plates were washed twice with 1X PBS, attached cells were harvested using warm TrypLE (Thermo Fisher Scientific—Waltham, MA), washed twice in PBS, and reconstituted at 5 × 10^6^ cells/ml in sterile saline and kept on ice, 100 µl of cells are used for i.v. injection.

### Isolation of monocytes from bone marrow (BMMs) and blood

Bone marrow was collected from femur and tibia using a 28G needle under sterile conditions and 1 ml of blood was collected in EDTA tubes, red blood cells lysed with ACK lysing buffer (ThermoFisher, Gaithersburg, MD, USA). Cells were strained, washed and resuspended in PBS/1 mM EDTA/2% FBS at 1 × 10^8^ cells/ml. Circulating peripheral-derived monocytes (PDM) from blood and BMMs were isolated using the EasySep™ Mouse Monocyte Kit #19861 (Stem cell technologies, Seattle, WA, USA) following manufactures protocol. Bone marrow cells 2.5 × 10^6^ cells/ml, and 7.5 × 10^5^ cells/ml from blood, were reconstituted in sterile saline and i.v. injected at 200 µl, single cells were cryopreserved in freezing media for scRNA sequencing or resuspended in 100 µl of TRIzol® for RNA extraction.

### Tissue harvesting, serial coronal sectioning and immunohistochemistry

Mice were anesthetized with 150 mg/kg ketamine and xylazine 15 mg/kg followed by transcardial perfusion with 20 ml cold 1XPBS followed by cold 4% buffered paraformaldehyde (pH 7.4) and tissue retrieval and processing as previously described [34, 61, 63, 64]. Immunohistochemistry was performed as previously described [34, 59, 60], and incubated with primary antibodies (goat anti-GFP, ThermoFisher; rabbit anti-Iba1, WAKO; rat Cd11b, Abcam; rabbit anti-CD45, Cell signaling). Images were acquired using a Nikon ECLIPSE Ti2 Inverted confocal microscope with a motorized stage and a Nikon C2 laser system (Nikon, Melville, NY).

### Evaluation of lesion volume and cell quantification by optical fractionator

Serial coronal sections were stained with Cresyl violet acetate (Electron Microscopy Sciences, Hatfield, PA). Lesion volume in mm^3^ was assessed by a blinded investigator using the Cavalieri Estimator from StereoInvestigator software (MicroBrightField, Williston, VT, USA) on an upright Olympus BX51TRF motorized microscope (Olympus America, Center Valley, PA, USA) and cell quantification by optical fractionator was performed as previously described [59, 60].

### RNA extraction, cDNA and quantitative real-time PCR

Total RNA was extracted from TRIzol® preserved samples using Direct-zol RNA Minipreps (Zymo, Irvine, CA). RNA quantification was carried out by ND-1000 (NanoDrop) then converted to cDNA using 1ug of RNA using iScript™ cDNA synthesis kit (Biorad, Hercules, CA) per manufacturer’s specifications. For qRT-PCR analysis, 1 µl of cDNA per 10 µl reaction was amplified using iTaq™ Universal SYBR® Green Supermix (Biorad, Hercules, CA). Expression changes were calculated using ΔΔCt method with reference to *Gapdh* and relative expression was calculated and normalized to WT or sham control sample. All primers were tested for primer efficiency and used at 80–105%.

### Protein analysis and cytokine array

Proteome Profiler Mouse Cytokine Array Kit, (#ARY006) was used to measure cytokines from 4 × 4 mm cortical samples homogenized in RIPA buffer with proteinase and phosphatase inhibitors. Quantification using RC DC™ Protein Assay Kit I #5000121 (Biorad, Hercules, California, USA) following manufacturer instructions. Membranes were imaged together in a ChemiDoc™ Imaging System. Images were analyzed using gels tools in FIJI software to quantify in duplicate wells, and compared relative to contralateral control.

### Fluorescence-activated cell sorting (FACS)

A 4 × 4 mm cortical tissue was microdissected in ice-cold L15 dissecting media (ThermoFisher, Waltham, MA) and subjected to papain neural dissociation (Miltenyi Biotec, Auburn, CA) per manufacturer instructions. Blood samples were prepared from 1 ml of cardiac puncture collected in EDTA, subjected to ACK lysing buffer, resuspended in 100ul PBS per sample and placed in a 96-well plate. Cells were incubated at 1/500 in Zombie aqua (BioLegend, San Diego, CA) for 20 m at room temperature, washed and centrifuged at 300*g* at 4 °C for 5 min. Cells were resuspended in PBS/ 1% FBS, washed then incubated with 2% FC blocker in 100ul of PBS/1% FBS/2 mM EDTA on ice for 10 min, followed by 20 m primary antibody incubation (APC-CD45; Alexa Fluor 700-CD11b; PE/Cy7- Ly6G; BV-421-Ly6C; Alexa Fluor 488-CD115; BV-650-CCR2; BioLegend, San Diego, CA). Unstained and single-color controls for each antibody target were included to set limits in flow analysis. BD FACSAria™ II Flow Cytometer was used to collect ~ 50k cells per sample and data analyzed using FlowJo v10 (BD, Ashland, Oregon).

### scRNA sequencing, library generation and Gene Ontology enrichment

Each sample containing 1 × 10^6^ cells with over 95% viability were cryopreserved in 1 ml of CryoStore® CS10 media (Stem cell technologies, Seattle, WA, USA). Cells were sent to Medgenome for scRNAseq (Foster City, California, USA). ScRNA-seq libraries were generated from the isolated monocytes using the Chromium Next GEM Single Cell 5ʹ v2 chemistry (10 × Genomics) and sequenced on a NovaSeq 6000 (Illumina), followed by generation of FASTQ files from BCL file using Cell Ranger V 7.0.0 (10 × Genomics) mkfastq analysis pipeline. GO enrichment with R package clusterProfiler(v4.4.4) and org.Mm.eg.db (v3.15.0) was used. The top 10 biological processes (BP) terms were used to generate GO circle plot with R package GOplot (v1.0.2). Enhanced volcano plot was generated based on DEGs, FC 1.2, adjusted *p*-value < 0.05.

### Analyses of scRNA-seq

Alignment of the libraries, and read counts were performed using Cell Ranger V7.0 (10X Genomics) using Mouse RNA-Seq Database as a reference. This database consists of normalized expression values of 358 bulk Mouse RNA-seq samples of sorted cell populations. Quality control and analysis were done using Seurat (V 4.1.0 Read10X function) [65]. Genes that are not expressed in at least 3 cells and cells that do not have a minimum of total 200 expressed genes were excluded and we filtered for doublets (DoubletFinder package, v2.0.3). Data were normalized by global-scaling normalization method “LogNormalize” and Seurat was used for clustering. We applied modularity optimization by Louvain algorithm or SLM to iteratively group cells, and visualize the data using UMAP. A subset of features that exhibit high cell-to-cell variation in the dataset were generated (i.e., they are highly expressed in some cells, and lowly expressed in others). FindAllMarkers function from Seurat was used and unbiased cell type recognition using SingleR and Cellenics, leveraged reference transcriptomic datasets of pure cell types to infer the cell of origin. The following markers were used for the clustering of classical (Ly6c, Ccr2, Ccr5, C5ar1, Cd88), non-classical (Cx3cr1, Nr4a1, Cebpb), neutrophil-like (Elane, Prtn3, Ctsg, MPO), MoDC (H2-Ab1, H2-Aa, Cd209a) monocytes subclasses.

#### LPS assay

Blood was collected with 0.5 M EDTA diluted at 1:10 and centrifuged at 300G for 6 min, the supernatant was collected and centrifuged at 14000G for 15 min before storage at − 80 °C. Samples were tested using the LPS ELISA kit (LSBio #LS-F55511) following manufacturers recommendation using 100 µl of undiluted plasma. Terminal cardiac blood collection was used for organism burden via plaque assay. Isolation of 100 µl of blood was immediately inoculated into a blood agar plate (Remel, Cat# R01215) and cultured for 7 days at 3 °C before colony enumeration.

#### Statistical analysis

All data were graphed using the GraphPad Prism program, version 9 (GraphPad Software, Inc., San Diego, CA). We used Student’s two-tailed t-test for comparison of two experimental groups and one-way or two-way ANOVA for multiple comparisons, were appropriate, followed by appropriate post hoc test. We identified changes as significant at *p* value < 0.05. Each mean value was reported alongside the standard error of the mean (SEM). All mice were coded and experimenters blinded to treatment conditions.

### Supplementary Information

**Additional file 1: Figure S1.** Dil-Liposome localizes in white blood cells, and is distributed in Iba1 + cells of liver and spleen, but not the brain. (A-B) Representative confocal images for brain cortex showing Dil-LPM (red) inside blood vessels (CD31 + , white) and not in microglia (Iba-1 + , green) or brain parenchyma. (E–G) Dil-LPM is not detected in the brain of naïve mice at 1 h (F) or 24 h after injection (G). (H) Representative confocal images showing Dil-LPM (red) staining in Iba1 + (green) cells in kidney, (I) liver parenchyma (J) and spleen. n = 5/group, *P < 0.05; **P < 0.01; ****P < 0.0001. One-way ANOVA with Bonferroni post hoc.

**Additional file 2: Figure S2.** Purity of BMMs and flow cytometry of ipsilateral cortex at 1dpi after i.v. GFP^+^ BMDM injection. (A) qRT-PCR show clear upregulation of monocytes markers Cxcr1 and CD209 and consistent downregulation of other bone marrow cell markers such as Ly6G, CD3, CD8, CD19a, CD4a, MPI, and VE-cad. (B) Flow cytometric analysis of infiltrating GFP-labeled BMDMs in cortical tissue from reconstituted CCI-injured or naïve mice at 1dpi. Cells gated for GFP show that ~ 10% of cortical cells in the damaged tissue were GFP labeled. GFP + cells are positive for Ccr2, Ccr2/Cx3cr1, and Ly6C monocyte/macrophage lineage markers.

**Additional file 3: Figure S3.** Influx of innate immune cells and mRNA expression alterations in the damaged cortex of control and Cl-LPM injured mice. (A, B) Representative confocal images for ipsilateral cortex of control-LPM (A) and Cl-LPM (B) treated mice immune-stained for Ly6G (green), CCR2 (red), and DAPI (blue) at 1dpi. Scale = 200 µm. (A1-5 and B1-5) High magnification images from the perilesional area of control and CL-LPM treated mice, respectively. Scale = 50 µm. (C-D) Flow histograms of CD45^hi^, CD11b^+^ microglia population are negative for Ly6G, CCR2 and Ly6C. (E) Non-biased stereology count of the estimated number of CD68^+^ cells and CD68^+^/TMEM119^+^ microglia. (F) Relative mRNA expression of *IL12, TGFb, IL-10, IL-4,* and *IL-6* in the ipsilateral cortex of control-LPM or CL-LPM treated mice at 1dpi. (n = 5–10 mice/group), *P < 0.05; **P < 0.01; ***P < 0.001; ****P < 0.00001.

**Additional file 4: Figure S4.** Single-cell sequencing of blood-enriched monocytes at 1dpi. (A-B) automated cell type annotation based on single-cell sequencing showed that blood PDMs from control LPM (A) and Cl-LPM (B) displayed a gene expression profile characteristic of monocytes, although there is a small percentage of B cells, dendritic cells, granulocytes, macrophages, T cells and NK cells also appear to be present in both samples (C-D).

**Additional file 5: Figure S5.** Effects of Cl-LPM on spleen and blood. (A) Spleen weight in grams was measured at 0, 7, 12 dpi in control and Cl-LPM treated CCI mice. (B) Representative H&E section of control and CL-LPM spleen showing cortical atrophy of the red pulp. (C) Smaller size can be seen in the gross images of spleen after CL-LPM compared to control LPM treatment at day 0 (naïve). (D) Iba-1 immunostaining shows prominent expression in control spleen that is drastically reduced in Cl-LPM mice. (E) Spleen weight is reduced at day 0 (naïve) and at 1 day post-sham and CCI injury. (F) Flow cytometry analysis of bone marrow cells from control or Cl-LPM treated mice at day 0, shows no significant change in CD115 + BM monocytes. (G) Blood culture for 7 days to detect microbial growth. (H) ELISA LPS detection in blood at 1dpi. *P < 0.05; **P < 0.01; ***P < 0.001; ****P < 0.00001. ns = not significant. Scale = 250 µm in B and D; Scale = 25 µm in B, D and Scale = 1 cm in C.

## Data Availability

Datasets analyzed during the study are available from the corresponding author on reasonable request.

## Abbreviations

Cl-LPM: Clodronate liposomes
CCI: Controlled cortical impact
TBI: Traumatic brain injury
dpi: Days post-injury
i.v.: Intravenous
BMDMs: Bone marrow-derived monocyte/macrophages
BMM: Bone marrow-derived monocytes
IHC: Immunohistochemistry

## Declarations

### Significance statement

Monocytes regulate tissue damage and repair in the brain. The effects of sterile brain injury on their phenotypic identity and recruitment, under emergency conditions were studied at the single-cell level. Our work has uncovered a novel immunoregulatory state that may be sufficient to provide neuroprotection.
