# Supplementary figures and images for: Immunoregulatory and neutrophil-like monocyte subsets with distinct single-cell transcriptomic signatures emerge following brain injury
Source: J Neuroinflammation. 2024 Feb 3;21:41. doi: 10.1186/s12974-024-03032-8 (PMC10838447; doi:10.1186/s12974-024-03032-8)

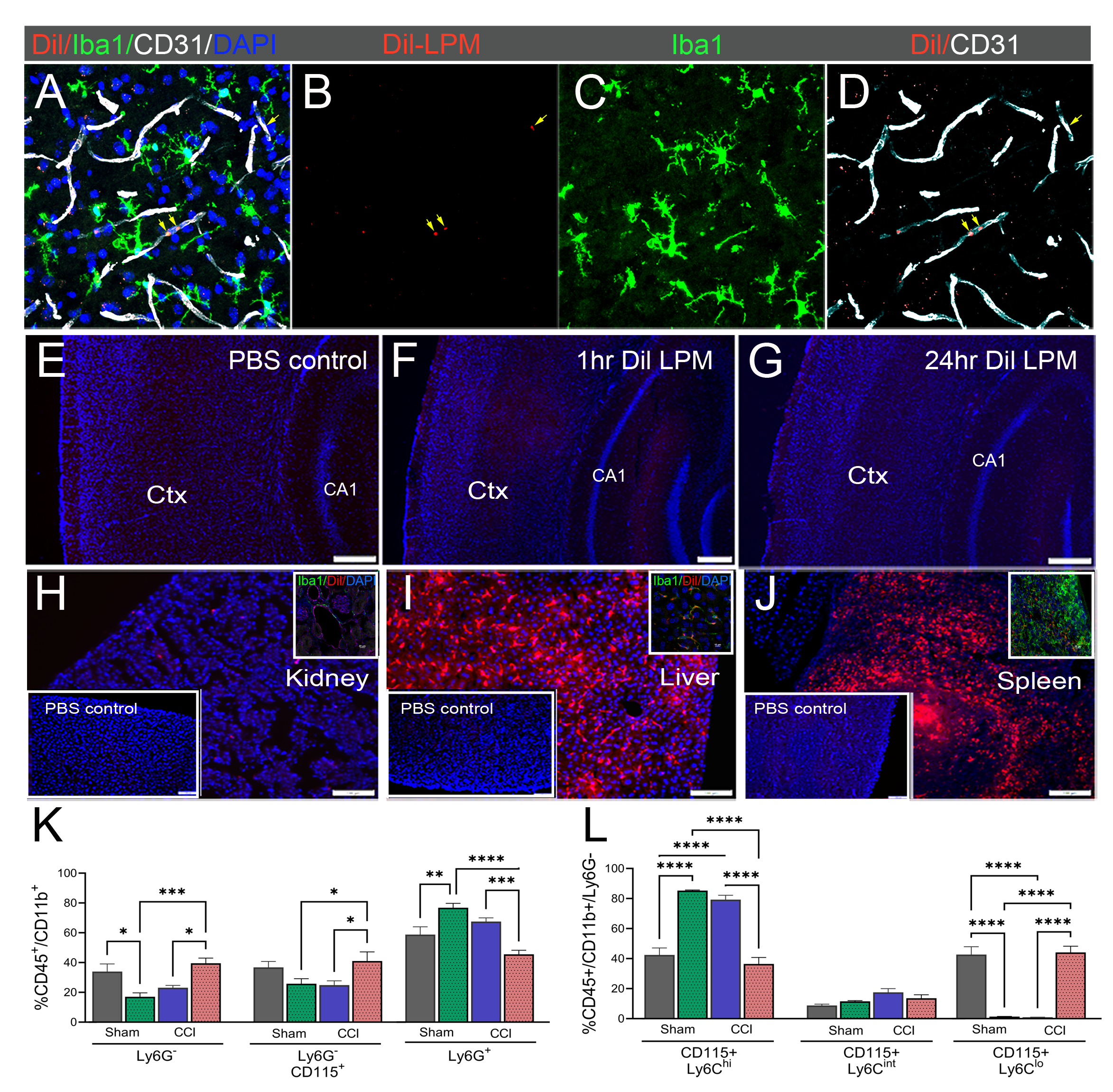

Supplement: Supplementary file 1 — Additional file 1: Figure S1. Dil-Liposome localizes in white blood cells, and is distributed in Iba1 + cells of liver and spleen, but not the brain. (A-B) Representative confocal images for brain cortex showing Dil-LPM (red) inside blood vessels (CD31 + , white) and not in microglia (Iba-1 + , green) or brain parenchyma. (E–G) Dil-LPM is not detected in the brain of naïve mice at 1 h (F) or 24 h after injection (G). (H) Representative confocal images showing Dil-LPM (red) staining in Iba1 + (green) cells in kidney, (I) liver parenchyma (J) and spleen. n = 5/group, *P < 0.05; **P < 0.01; ****P < 0.0001. One-way ANOVA with Bonferroni post hoc. [file 12974_2024_3032_MOESM1_ESM.tif]

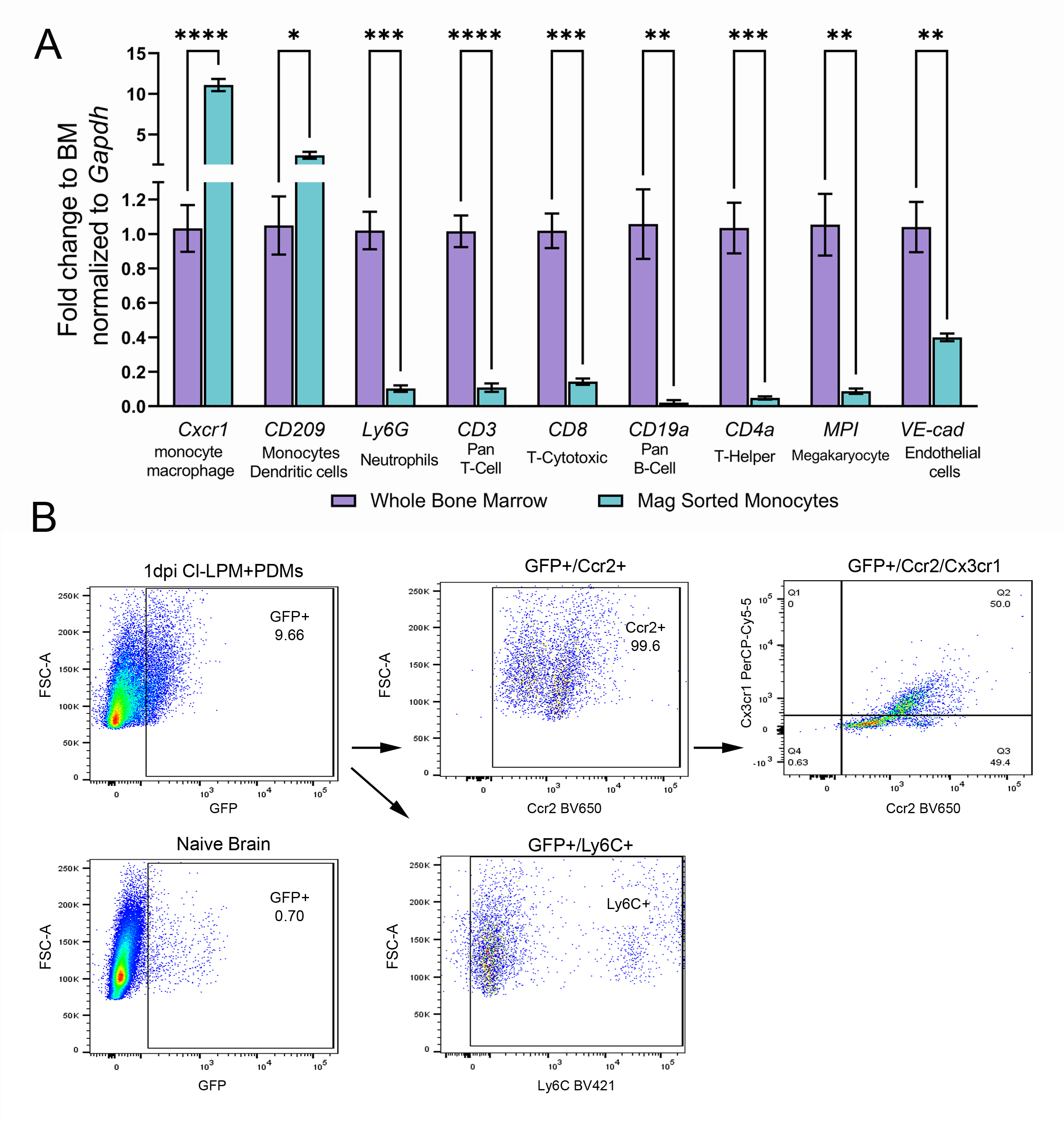

Supplement: Supplementary file 2 — Additional file 2: Figure S2. Purity of BMMs and flow cytometry of ipsilateral cortex at 1dpi after i.v. GFP+ BMDM injection. (A) qRT-PCR show clear upregulation of monocytes markers Cxcr1 and CD209 and consistent downregulation of other bone marrow cell markers such as Ly6G, CD3, CD8, CD19a, CD4a, MPI, and VE-cad. (B) Flow cytometric analysis of infiltrating GFP-labeled BMDMs in cortical tissue from reconstituted CCI-injured or naïve mice at 1dpi. Cells gated for GFP show that ~ 10% of cortical cells in the damaged tissue were GFP labeled. GFP + cells are positive for Ccr2, Ccr2/Cx3cr1, and Ly6C monocyte/macrophage lineage markers. [file 12974_2024_3032_MOESM2_ESM.tif]

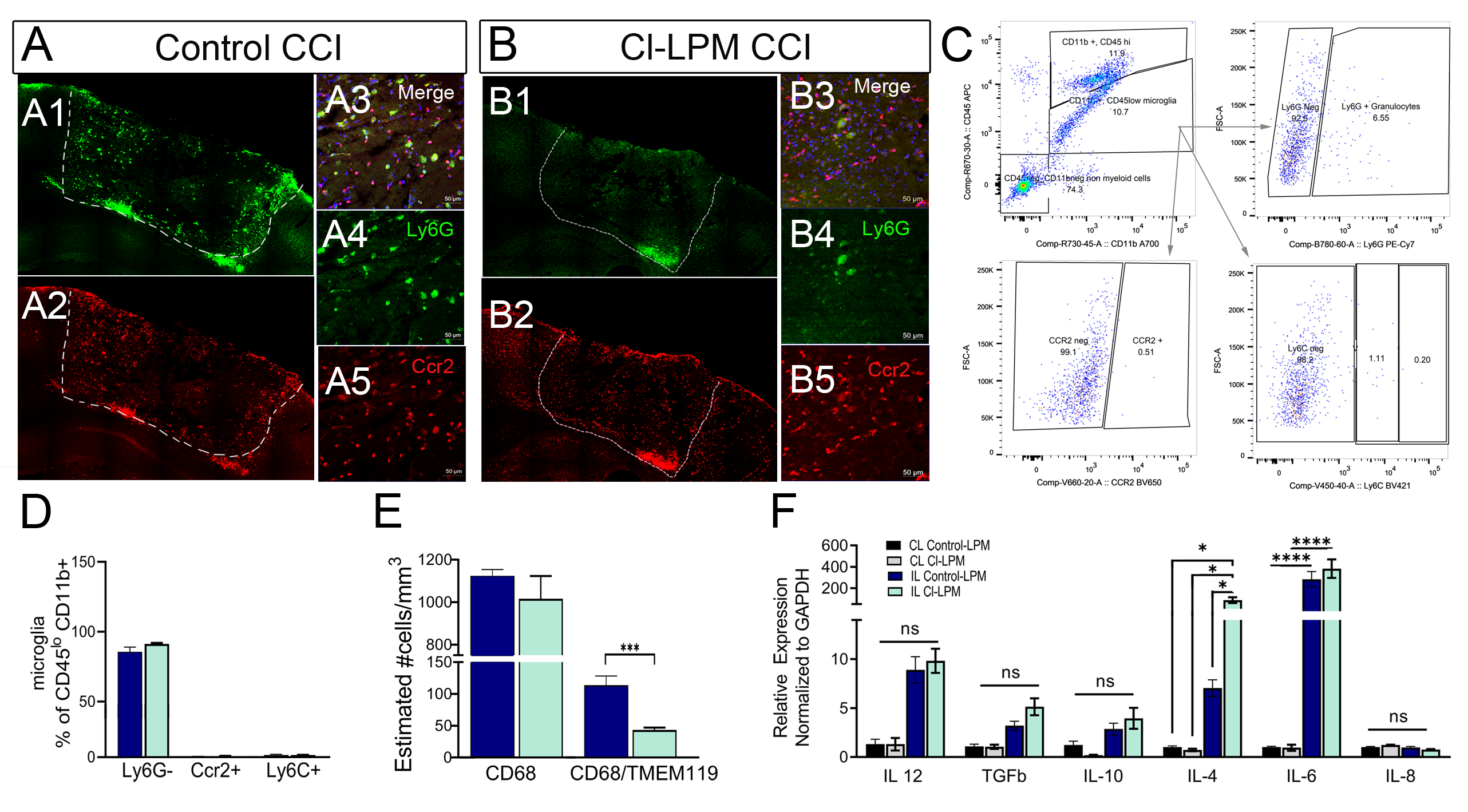

Supplement: Supplementary file 3 — Additional file 3: Figure S3. Influx of innate immune cells and mRNA expression alterations in the damaged cortex of control and Cl-LPM injured mice. (A, B) Representative confocal images for ipsilateral cortex of control-LPM (A) and Cl-LPM (B) treated mice immune-stained for Ly6G (green), CCR2 (red), and DAPI (blue) at 1dpi. Scale = 200 µm. (A1-5 and B1-5) High magnification images from the perilesional area of control and CL-LPM treated mice, respectively. Scale = 50 µm. (C-D) Flow histograms of CD45hi, CD11b+ microglia population are negative for Ly6G, CCR2 and Ly6C. (E) Non-biased stereology count of the estimated number of CD68+ cells and CD68+/TMEM119+ microglia. (F) Relative mRNA expression of IL12, TGFb, IL-10, IL-4, and IL-6 in the ipsilateral cortex of control-LPM or CL-LPM treated mice at 1dpi. (n = 5–10 mice/group), *P < 0.05; **P < 0.01; ***P < 0.001; ****P < 0.00001. [file 12974_2024_3032_MOESM3_ESM.tif]

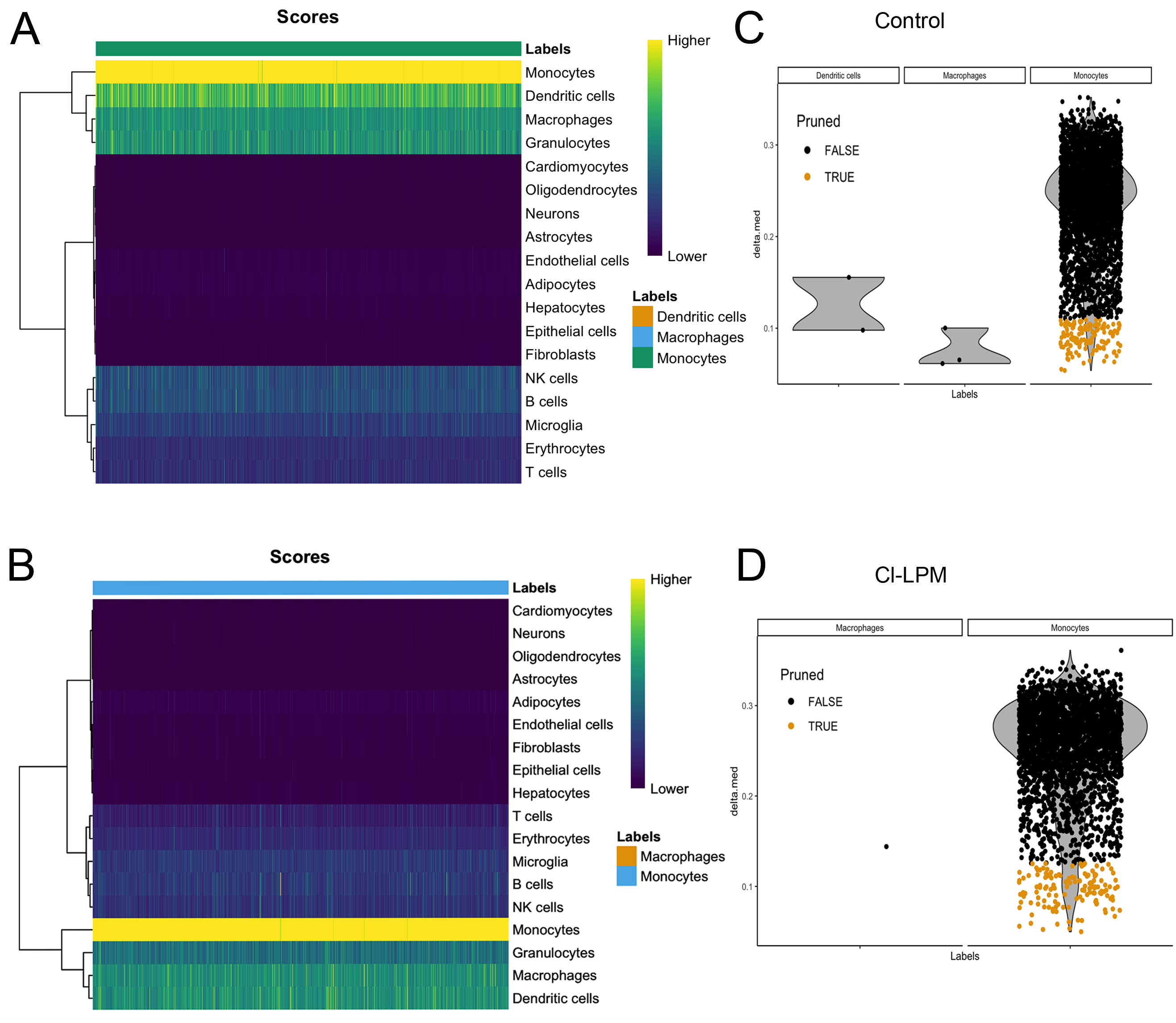

Supplement: Supplementary file 4 — Additional file 4: Figure S4. Single-cell sequencing of blood-enriched monocytes at 1dpi. (A-B) automated cell type annotation based on single-cell sequencing showed that blood PDMs from control LPM (A) and Cl-LPM (B) displayed a gene expression profile characteristic of monocytes, although there is a small percentage of B cells, dendritic cells, granulocytes, macrophages, T cells and NK cells also appear to be present in both samples (C-D). [file 12974_2024_3032_MOESM4_ESM.tif]

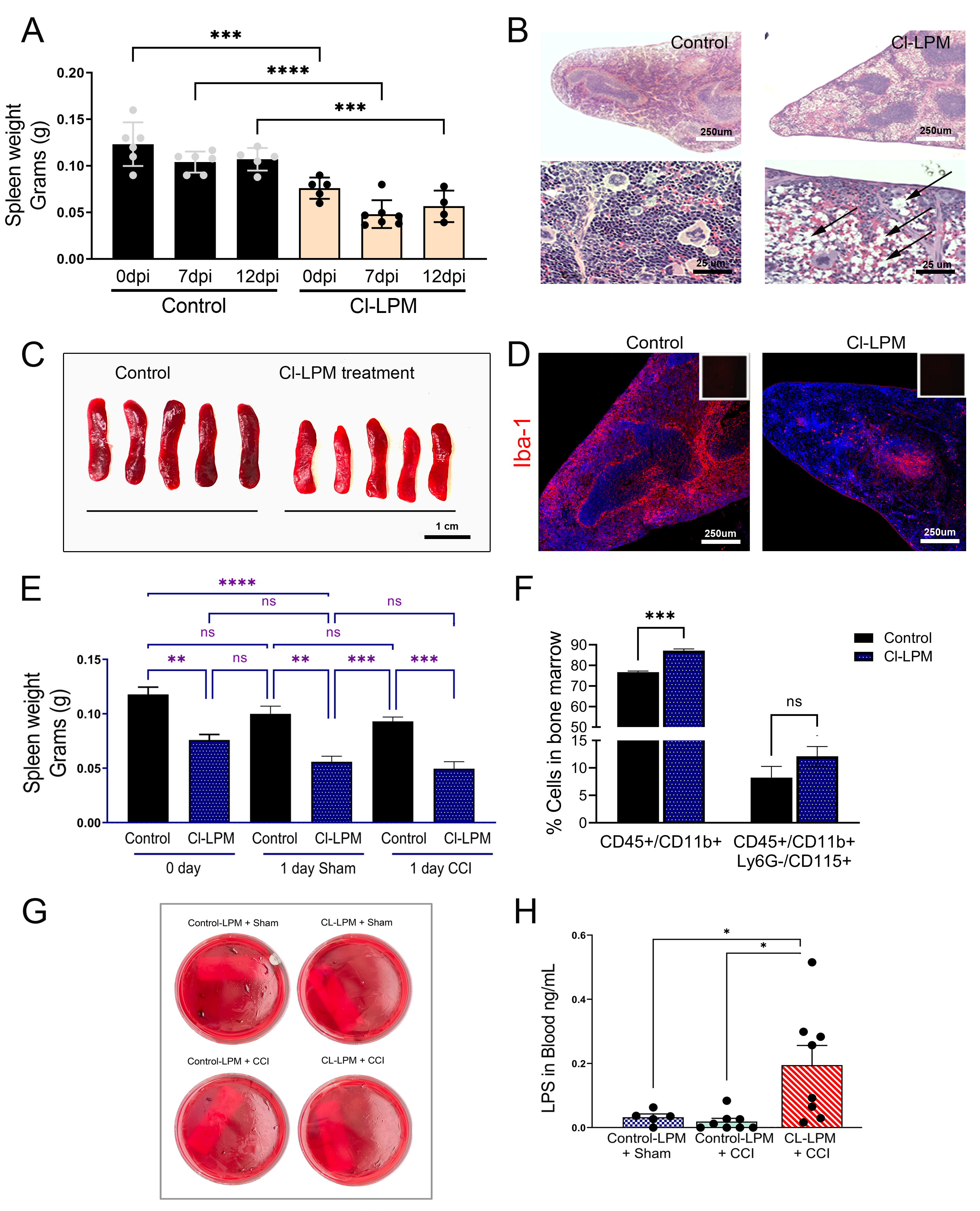

Supplement: Supplementary file 5 — Additional file 5: Figure S5. Effects of Cl-LPM on spleen and blood. (A) Spleen weight in grams was measured at 0, 7, 12 dpi in control and Cl-LPM treated CCI mice. (B) Representative H&E section of control and CL-LPM spleen showing cortical atrophy of the red pulp. (C) Smaller size can be seen in the gross images of spleen after CL-LPM compared to control LPM treatment at day 0 (naïve). (D) Iba-1 immunostaining shows prominent expression in control spleen that is drastically reduced in Cl-LPM mice. (E) Spleen weight is reduced at day 0 (naïve) and at 1 day post-sham and CCI injury. (F) Flow cytometry analysis of bone marrow cells from control or Cl-LPM treated mice at day 0, shows no significant change in CD115 + BM monocytes. (G) Blood culture for 7 days to detect microbial growth. (H) ELISA LPS detection in blood at 1dpi. *P < 0.05; **P < 0.01; ***P < 0.001; ****P < 0.00001. ns = not significant. Scale = 250 µm in B and D; Scale = 25 µm in B, D and Scale = 1 cm in C. [file 12974_2024_3032_MOESM5_ESM.tif]
